# Tracing and Capturing the Epiblast Pluripotency of Sheep Preimplantation Embryos

**DOI:** 10.1002/advs.202417764

**Published:** 2025-06-30

**Authors:** Jinying Zhang, Runbo Li, Ruijie Luo, Qiang Zhang, Zimo Zhao, Haishen Xu, Minglei Zhi, Wenjie Jiang, Meng Wang, Xinze Chen, Zhiqiang Feng, Yingjie Wang, Yuhan Yang, Pengcheng He, Hanyue Su, Tianzhi Chen, Shunxin Wang, Yixuan Yao, Jinghui Yang, Fan Zhao, Tingting Li, Haitao Wang, Xiaosheng Zhang, Jinlong Zhang, Qiuyue Liu, Shuai Gao, Yuchang Yao, Jianyong Han, Suying Cao

**Affiliations:** ^1^ State Key Laboratory of Animal Biotech Breeding Frontiers Science Center for Molecular Design Breeding (MOE) College of Biological Sciences China Agricultural University Beijing 100193 China; ^2^ Animal Science and Technology College Beijing University of Agriculture Beijing 102206 China; ^3^ Institute of Cardiovascular Diseases Xiamen Cardiovascular Hospital School of Medicine Xiamen University Xiamen 361006 China; ^4^ College of Animal Science and Technology Northeast Agricultural University Harbin 150038 China; ^5^ State Key Laboratory of Molecular Developmental Biology Institute of Genetics and Developmental Biology Chinese Academy of Sciences Beijing 100101 China; ^6^ Institute of Animal Sciences and Veterinary Tianjin Academy of Agriculture Sciences Tianjin 300380 China; ^7^ State Key Laboratory of Animal Biotech Breeding Frontiers Science Center for Molecular Design Breeding (MOE) College of Animal Science and Technology China Agricultural University Beijing 100193 China

**Keywords:** embryo development, epiblast, pluripotency, sheep, single‐cell RNA seq

## Abstract

Capturing different pluripotent state stem cells from epiblast in vitro helps understand embryonic development and provides invaluable cell sources for basic research and regenerative medicine. Sheep are not only one of the most important livestock species in agriculture but also serve as an ideal preclinical model for studying human disease. Single‐cell transcriptome analysis of sheep preimplantation embryos from embryonic day (E) 1 to E14 is performed to investigate the pluripotency changes of epiblast and elucidate the pluripotent regulation signaling. By combination of growth factors or inhibitors of JAK/STAT3, FGF, WNT, and TGF‐β pathways in the culture medium, sheep formative and primed pluripotent stem cells (sfPSCs and spPSCs) are established respectively. The newly derived PSCs could maintain over 100 passages and differentiate into three germ layers. In addition, sfPSCs and spPSCs exhibit different molecular features, and sfPSCs have the ability of contribution to ICM and can be used as donor cells for producing cloned embryos efficiently. A cross‐species comparison of early embryo development in mouse, pig and sheep illustrates the conservation of the epiblast naïve to primed state transition process and the divergence of the developmental events time points, the specific gene expression patterns and pluripotent regulation signaling. These studies are expected to improve our understanding of mammal early embryo development and present a reference for defining pluripotency.

## Graphical abstract

This study performs a comprehensive single‐cell transcriptome analysis of sheep embryos from embryonic (E) day 1 to E14 to elucidate the mechanism of early lineage specification and the pluripotency changes (naïve, formative, and primed) of epiblast. Based on scRNA‐Seq data, culture conditions are identified and sheep stable formative and primed pluripotent stem cells (sfPSCs and spPSCs) are derived in vitro for long‐term culture. This work provides valuable data resources to investigate sheep early embryo development and broaden our understanding of livestock PSCs.

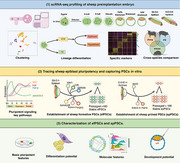

## 1 Introduction

Embryonic epiblast pluripotency spans a dynamic continuum developmental progression of naïve, formative and primed states, which is the source of pluripotent stem cells (PSCs).^[^ ^1^, ^2^ ^]^ In vitro cultured PSCs reflect the epiblast pluripotency seen in vivo, and different culture conditions contribute to distinct pluripotency states.^[^ ^3^, ^4^ ^]^ To date, stable naïve, formative and primed PSCs have been established in mice, humans and monkeys, exhibiting several differences in signal requirements, gene regulatory network, epigenetics and metabolism.^[^ ^5^, ^6^, ^7^, ^8^, ^9^, ^10^, ^11^, ^12^, ^13^ ^]^ Due to ethical considerations, the full developmental potential of naïve human PSCs can not be determined.^[^ ^14^ ^]^

Livestock PSCs are expected to facilitate biomedical and accelerate animal breeding in agriculture.^[^ ^15^, ^16^, ^17^ ^]^ Current studies using high throughput single‐cell transcriptomics deepen the understanding of early embryo development and strongly affect the establishment of PSCs.^[^ ^18^, ^19^, ^20^, ^21^, ^22^ ^]^ Guided by single‐cell RNA sequencing analysis of the pig preimplantation embryo, a 3iLAF culture medium was defined to stabilize pig pre‐gastrulation epiblast stem cells (pgEpiSCs) for long‐term passage.^[^ ^23^ ^]^ Similar gene expression patterns and signalings of the epiblast lineage development between bovine and pig also support the generation of bovine epiblast stem cells (bEpiSCs) in analogous culture conditions.^[^ ^24^ ^]^ Transcriptome analysis reveal that the molecular features of pgEpiSCs and bEpiSCs strongly resemble formative epiblasts.^[^ ^23^, ^24^ ^]^

Sheep are docile ruminants and widely utilized for the production of wool and meat. Relative to other livestock species used for biological research, sheep and their fetuses are similar in size to humans, making them as ideal experimental animals model for studying human fetal disease and improving therapeutic strategies.^[^ ^25^, ^26^ ^]^ Sheep PSCs are also valuable cell resources for applications in various fields.^[^ ^27^, ^28^ ^]^ Research on sheep PSCs began early in the 1990s,^[^ ^29^, ^30^ ^]^ but the pace is slow due to lack of in‐depth research on the regulatory mechanisms of preimplantation embryogenesis and the dynamic regulation networks of epiblast pluripotency.^[^ ^31^, ^32^ ^]^ The reported sheep induced pluripotent stem cells (iPSCs) have not shown silencing of exogenous genes,^[^ ^33^, ^34^ ^]^ which limits the application of sheep PSCs in the fields of agriculture and biomedicine. For decades, researchers have faced challenges in deriving stable sheep PSCs with defined pluripotent states that can be cultured long‐term in vitro.

Recently, sheep PSCs can be derived using CTFR medium and AFX medium.^[^ ^35^, ^36^ ^]^ These PSCs could maintain colony morphology, expressing classic pluripotent genes and the transcriptional features showed primed like states. However, the pluripotency of these PSCs have not been fully characterized, and sheep PSCs with other defined pluripotent states resembled the epiblast have not been established. Therefore, there is an urgent need to elucidate the regulatory mechanism of the epiblast pluripotency in sheep, and the long‐term suitable culture system for defined pluripotency of sheep PSCs requires further exploration and refinements.

This study aimed to depict a comprehensive single‐cell transcriptome atlas of sheep preimplantation embryos from E1‐E14 to trace the lineage development and profile the molecular basis of the epiblast pluripotency changes. Based on pluripotency signaling analysis of the epiblast, we developed different culture conditions for capturing stable formative PSCs from E8‐E11 epiblasts and stable primed PSCs from E12‐E14 epiblasts. The establishment of stable sheep PSCs holds prospects for their applications in basic research, gene edited animal models construction and stem cell based breeding. The dataset resources are valuable for investigating the differences and conservativeness of embryo development among species and provide references for evaluating PSCs pluripotent states.

## 2 Results

### 2.1 Single‐Cell Transcriptomes Reveal the Zygote Genome Activation and Lineage Segregation During Sheep Embryo Development

**Figure 1:**
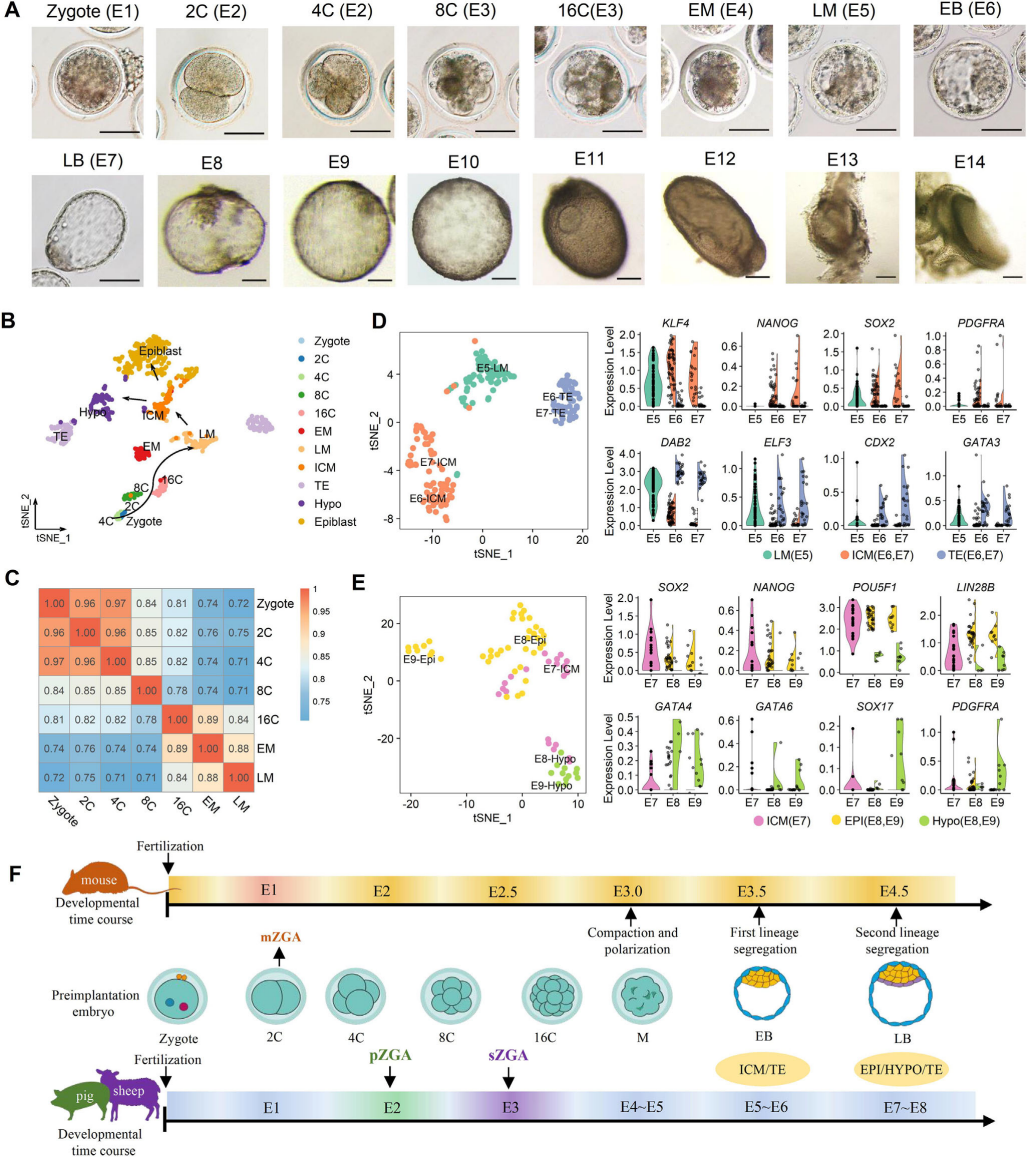
Single‐cell transcriptional profiling reveals lineage segregation in sheep embryos. A) Collection of sheep embryos for single cell RNA‐ seq from embryonic day (E) 1 to E14. EM: early morula, LM: late morula, EB: early blastocyst, LB: late blastocyst. Scale bar for E1‐E8, 100 µm; scale bar for E9‐E14, 500 µm. B) t‐SNE plots of all sheep embryonic cells, colors indicate different developmental stages and cell types. Arrows represent the developmental trajectories of embryonic lineages. EPI: epiblast, HYPO: hypoblast, ICM: inner cell mass, TE: trophectoderm. C) Heatmap representation of transcriptome correlation dynamics during the zygotic genome activation (ZGA) process. The values indicate Spearman's correlation coefficients of gene expression levels from zygote to late morula stage. D) t‐SNE plot shows the cluster of E5‐E7 stage embryonic cells during the first lineage differentiation. The violin plot displays the differential expression of key lineage marker genes. E) t‐SNE plot indicates the lineage separation of EPI and HYPO at embryonic stages E7‐E9, key lineage marker genes are shown in violin plots. F) Schematic diagram showing the developmental time point comparison of ZGA, lineage differentiation process of mouse, pig and sheep preimplantation embryo.

To trace the development trajectories of zygote genome activation (ZGA) and lineage segregation process during sheep embryo development, we established a detailed single‐cell transcriptome analysis of sheep in vivo embryo from embryonic day (E) 1 to E14 (**Figure** **1**A). Totally, 1,092 single cells were separated from 60 embryos and sequenced. After filtering out low‐quality and outlier cells, we retained 724 single‐cell transcriptomes for subsequent analysis (Figure S1A and Table S1, Supporting Information). According to the developmental stage and specific lineages based on known markers, we grouped the cells into different cell types by t‐distributed stochastic neighbor embedding (t‐SNE), which provided a clear visualization of the embryo development trajectory (Figure 1B; Figure S1B, Supporting Information). Subsequently, based on the results of Weighted Gene Co‐expressed Network Analysis (WGCNA), the cells were identified to several modules most strongly associated with distinct lineage cell types. Representative marker genes and key GO/KEGG pathway enrichments of each module further support the distinct cell assignment (Figure S1C and Table S2, Supporting Information).

Zygote genome activation marked by significant changes in gene expression patterns and transcription levels, which set the stage for embryo development.^[^ ^37^, ^38^ ^]^ In sheep embryos, we observed the transition from 8‐cell to 16‐cell stage exhibited significant transcriptomic correlation alterations (Figure 1C). To understand how ZGA initiation varies among species, we analyzed the gene expression trend of a series of conserved maternal genes, ribosomal genes, and splicing factor genes, verifying that ZGA occurs at the 2‐cell stage in mice, 4‐8‐cell stage in humans and pigs, consistent with previous reports (Figure S2A, Supporting Information).^[^ ^39^, ^40^, ^41^ ^]^

According to the developmental stage and specific lineage genes, dimensionality reduction clearly shows the lineage segregation process (Figure 1D,E). In the late morula (LM) stage at E5, although t‐SNE did not clearly distinguish two cell clusters, some cells exhibited heterogeneous expression of classic precursor marker genes for ICMs (e.g., *KLF4*, *SOX2*, *PDGFRA*) or TEs (e.g., *DAB2*, *ELF3*, *GATA3*), which indicated that the first lineage segregation of sheep embryo was beginning. In addition, the expression of *CDX2* and *NANOG* was detected only in a few pre‐TEs or pre‐ICMs at this early stage, but *POU5F1* expression was seen in all cells (Figure S2B, Supporting Information). This feature is conservative with the observations in pig, bovine and human embryo ICM/TE development.^[^ ^24^, ^42^, ^43^ ^]^ At the E6 early blastocyst stage, the ICMs and TEs were distinctly identified into two populations, with upregulation of *KLF4*, *NANOG* in ICMs and *CDX2*, *ELF3* in TEs, which suggests that the first lineage specification of ICM/TE is completed (Figure 1D; Figure S2C, Supporting Information). Next, we found a few cells with heterogeneous expression of *GATA6*, *GATA4*, *SOX17* and *NANOG* in E7 ICMs, implying the beginning of the ICMs‐derived hypoblast segregation (Figure S2D, Supporting Information). At E8 and E9, *GATA4* and *NANOG* positive cells were gradually divided into two populations (Figure S2E,F, Supporting Information), indicating the end of the second lineage segregation. Eventually, the embryos established epiblast with the expression of *POU5F1*, *SOX2*, *NAONG*, and hypoblast with the expression of *GATA6*, *GATA4*, *SOX17*, and *PDGFRA* (Figure 1E).

Trophoblast is critical for conceptus elongation, attachment, and fetal‐maternal communication before implantation.^[^ ^44^ ^]^ Different from rodents and primates, the unique features of sheep, bovine, and pig are the embryos do not attach to the uterus endometrium immediately, and the trophoblast undergoes extensive elongation after blastocyst hatching.^[^ ^45^, ^46^, ^47^, ^48^ ^]^ The spherical embryo proliferates rapidly and changes its shape to ovoid and tubular from E10‐E14. We further analyze the transcription factor expression across different stages or lineages in sheep embryos (Figure S3A, Supporting Information). Notably, in our scRNA‐seq data, t‐SNE revealed two distinct TE clusters, which indicates significant transcriptomic changes in TEs development. To investigate the molecular characteristics of the two TE populations, we identified two subgroups of early TEs for E6‐E8 and late TEs for E9‐E14 (Figure S3B, Supporting Information). All TE cells exhibited high expression levels of classical marker genes of *CDX2*, *GATA3* (Figure S1C, Supporting Information). Compared with the late TEs, we found *KRT18*, *KRT8*, *DAB2*, *TP53*, and *TFAP2C* were highly expressed in early TEs (Figure S3C, Supporting Information), which enriched in GO terms including energy metabolism, establishment of cell polarity, regulation of cytoskeleton organization, regulation of epithelial cell migration and embryo implantation (Figure S3D and Table S3, Supporting Information). Meanwhile, we found *IGF2*, *CD63*, *ELF3*, *ITGA6*, *LRP2*, and *IFNT6* specific highly expressed in late TEs (Figure S3C, Supporting Information). Particularly *IFNT6*, which is known as the signal of maternal pregnancy recognition in most ruminants.^[^ ^49^, ^50^ ^]^ GO term enrichment revealed that the cell cycle‐related genes were upregulated, suggesting that rapid cell proliferation may contribute to late TEs development. Moreover, NFKB signaling is suggested to be required for embryo implantation also enriched in late TEs (Figure S3D, Supporting Information).^[^ ^51^ ^]^ These data provide important evidence for the functional difference between early TEs and late TEs in sheep embryo initial elongation and implantation.

Taken together, our scRNA‐seq data identified cells from E1‐E14 preimplantation stages of sheep embryos, which delineated the process of embryonic genome activation and the lineage differentiation trajectory of each germ layer. We also show a comparative diagram of mouse, pig and sheep preimplantation embryo development time course, which indicate the key developmental event time point was different among species (Figure 1F).

### 2.2 Tracing the Changes of Pluripotency During Sheep Epiblast Development

**Figure 2:**
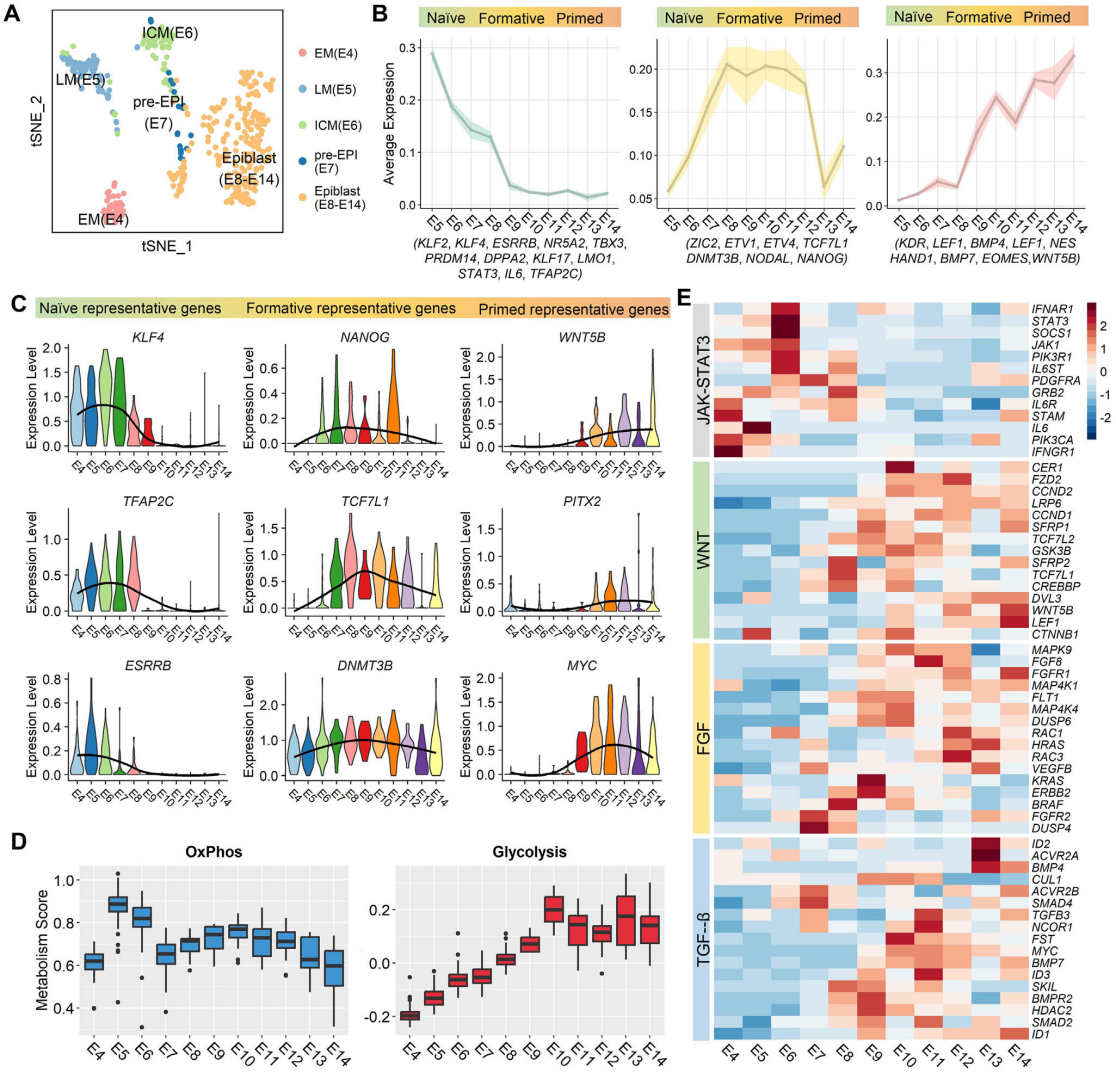
Tracing the pluripotent changes of sheep epiblast development. A) t‐SNE plots depict the cluster of sheep epiblast lineage cells at the developmental stage of E4‐E14. B) Line plots showing changes in expression levels of representative gene sets across naïve, formative and primed pluripotency states in the sheep epiblast lineage from E5 to E14, with shaded regions indicating confidence intervals. C) Violin plots showing the expression of selected naïve, formative and primed pluripotency specific genes in the EPI lineage. D) Box plots showing the expression trends of oxidative phosphorylation and glycolysis module genes in sheep epiblast lineage. The central line of each box represents the median (50th percentile), while the lower and upper edges correspond to the first (25th percentile) and third (75th percentile) quartiles, respectively. E) Expression changes of genes associated with JAK/STAT3, WNT, FGF, and TGF‐β signaling pathways in EPI lineage from E4 to E14.

To study the changes in sheep epiblast pluripotency and elucidate the core regulatory signaling pathways, we select E4 early morula, E5 late morula, E6 ICMs, E7 pre‐EPIs, E8‐E14 EPI cells for re‐clustering and subsequent analysis (**Figure** **2**A). We know epiblast development is accompanied by naïve‐to‐primed pluripotency transition in other species. To investigate whether the different pluripotent states exist in sheep epiblasts, we selected a list of known marker genes to speculate the progression of pluripotency trends and found that sheep epiblasts lost naïve pluripotency quickly from E5‐E7. E8 may be a critical point before the pre‐EPIs transition to the formative state. The formative pluripotency maintained a relatively long time from E8‐E11 and then shifted to the primed state (Figure 2B). Strictly, we observed the expression of representative naïve genes (e.g., *ESRRB*, *KLF4*, *IL6*, *IL6ST*, *DPPA2*, *TBX3*, *MAX*, and *TFAP2C*) was significantly decreased in cells from E5 ICMs to E7 pre‐EPIs (Figure 2C; Figure S4A, Supporting Information). Formative state marker genes (e.g., *ZIC2*, *ETV1*, *USP44*, *TCF7L1*, *LIN28B*, *NANOG*, *FSTL1*, and *DNMT3B*) exhibited relatively high expression from E8 to E11 EPIs, and the expression of classical primed pluripotent genes associated with gastrulation (e.g., *KDR*, *LEF1*, *MEST*, *CER1*, *VCAN*, *KRT19* and *WNT5B*) were subsequently upregulated from E12 EPIs (Figure 2C; Figure S4A, Supporting Information). In addition, metabolism gene module score analysis highlighted a metabolic switch from oxidative phosphorylation (peaking during the early blastocyst stages of E5 and E6) to glycolysis (peaking during the epiblast stage after E10) in sheep epiblast development (Figure 2D), which was conservative among species.^[^ ^52^, ^53^, ^54^ ^]^ The analysis of the gene interaction network of DEGs further demonstrates the different energy metabolisms requirement in epiblast lineage development (Figure S4B, Supporting Information).

Our insights into the changes in pluripotency regulation during sheep epiblast development is aimed to select suitable growth factors and signaling inhibitors for capturing the epiblast pluripotency and deriving PSCs in vitro. We analyzed the gene expression trends of pluripotency‐related pathways, including JAK/STAT3, FGF, TGF‐β, and WNT signaling. We found the genes involved in JAK/STAT3 signaling pathway (e.g., *IL6ST*, *STAT3*, *JAK1*, *IL6*, *SOCS1*, and *IL6R*) were highly expressed from E4 early morula to E7 pre‐EPIs but declined after E8 (Figure 2E). Pathway analysis highlighting the importance of JAK/STAT3 to maintain naïve pluripotency. Interestingly, *LIF* and *LIFR* expression were not detected in ICMs. Instead, *IL6*, *IL6R* and the co‐receptor *IL6ST* were highly expressed in ICMs, which suggests that *IL6* likely activates the JAK/STAT3 pathway by binding to its cognate receptor. Furthermore, we noted that WNT signaling‐related genes (e.g., *TCF7L1*, *TCF7L2*, *SFRP2*, *LRP6*, and *CREBBP*) gradually start to express at E8 EPIs. Other WNT family members activity associated with gastrulation and morphogenesis (e.g., *CER1*, *FZD2*, *CCND1*, *DVL3*, *WNT5B*, and *LEF1*) significantly increased during the process of the epiblast transition from formative to primed pluripotent state (Figure 2E). Therefore, inhibition of WNT signaling may be required for the derivation and maintenance of stable sheep epiblast stem cells. Recent research also showed that WNT inhibition conditions were effectively used in livestock PSCs maintenance.^[^ ^24^, ^55^, ^56^ ^]^ In addition, FGF signaling pathway‐related genes, such as *FGFR1*, *FGFR2*, *BRAF*, *DUSP6*, *VEGFB*, *MAPK9*, and TGF‐β signaling pathway‐related genes of *TGFB3*, *ACVR2B*, *ID3*, *ID1*, *SMAD2*, *BMPR2* exhibited sustained expression from E8 to E14, suggested that the cell proliferation and pluripotency maintenance require activating FGF and TGF‐β pathways (Figure 2E).

### 2.3 Cross‐Species Comparison of Epiblast Pluripotency among Mouse, Pig and Sheep

**Figure 3:**
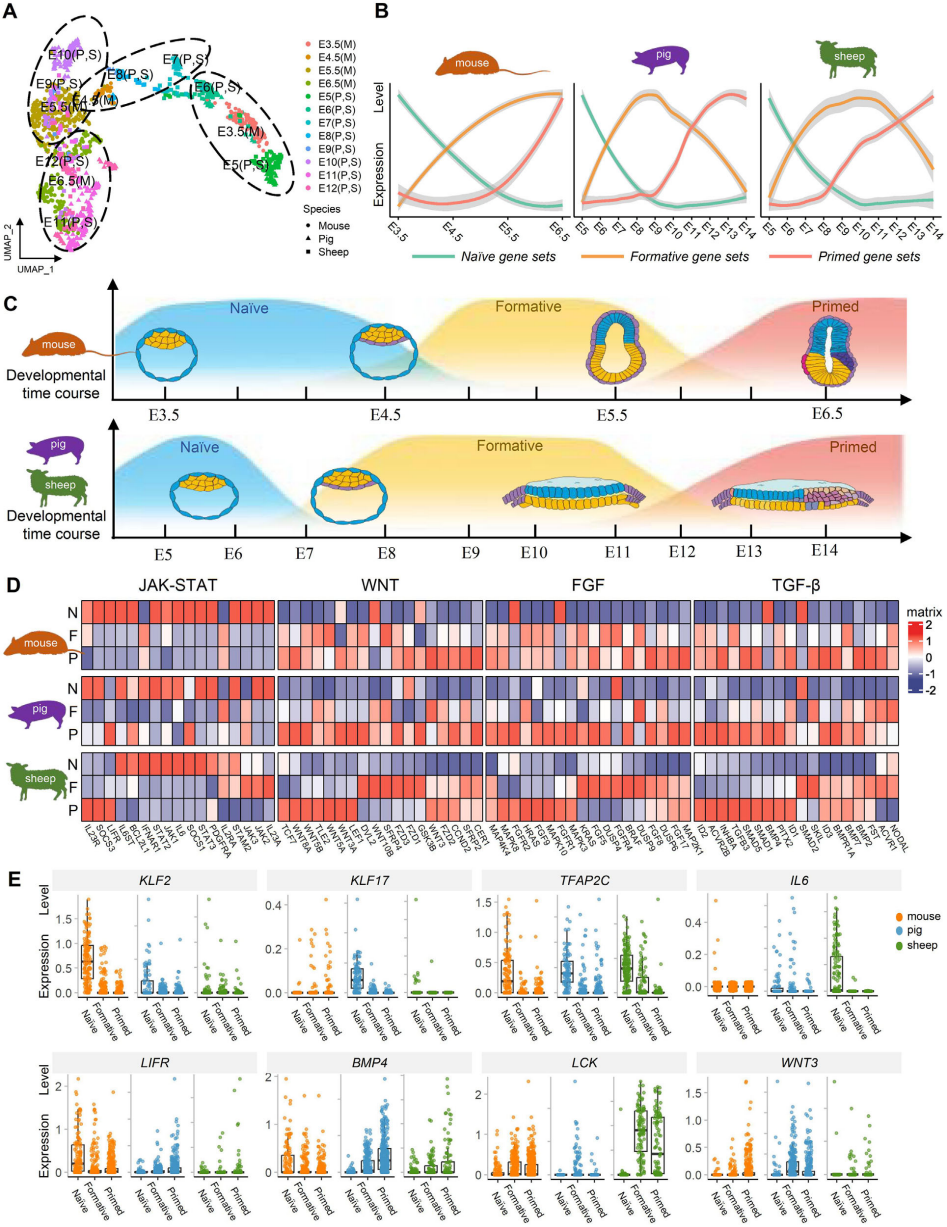
Cross‐species comparison of epiblast lineage development. A) The UMAP plot presents an integrated cross‐species cluster comparison of EPI lineage among sheep (E5‐E12), mouse (E3.5‐E6.5) and pig (E5‐ E12), with colors and shapes distinguishing different embryonic days and species. B) A time‐course expression plot with LOESS‐smoothed curves and confidence bands illustrates the average expression dynamics of pluripotency‐related gene sets across developmental stages. Gene expression profiles were used to infer pluripotency states of epiblast development. Naïve state markers (*KLF2*, *KLF4*, *NR5A2*, *TFAP2C*, *TFCP2L1*, *TBX3*, *ESRRB*, *KLF5*, *IL6ST*, *PRDM14*, *KLF17*, *STAT3*, *DPPA5*, *NROB1*, *DPPA2*, *PECAM1*) are shown in green, formative markers (*OTX2*, *TCF7L1*, *ETV4*, *ETV1*, *ZIC2*, *DNMT3B*, *DNMT3A*, *FGF5*, *LIN28B*, *WNT3*, *HESX1*) are shown in yellow, and primed markers (*EOMES*, *MIXL1*, *PITX2*, *TBXT*, *FOXA2*, *CER1*, *GATA4*, *LEF1*, *KDR*, *BMP4*, *BMP7*, *FGFR1*, *HAND1*, *SHH*, *WNT5A*, *WNT5B*, *WNT7B*, *WNT9A*, *MYC*, *KRT19*, *VCAN*, and *NES*) are shown in red. C) Schematic diagram comparing the pluripotency developmental coordinate spectrum of mouse, pig, and sheep epiblast. D) Cross‐species heatmap analysis of representative signaling pathway genes in mouse, pig, and sheep. Developmental stage classification (N: naïve, F: formative, P: primed): mouse — N (E3.5–E4.5), F (E4.5–E5.5), P (E5.5–E6.5); pig and sheep — N (E5–E7), F (E8–E11), P (E12–E14). E) Box plots showing the expression levels of representative genes at different pluripotency stages among species.

Cross‐species comparisons of epiblast at different developmental stages help us understand the conservative and differential signaling regulations during pluripotency transition and species‐specific features, which can provide reference for us to derive stable PSCs of sheep. Here, we conducted a comparative transcriptome analysis using two published datasets of mouse and pig embryos,^[^ ^23^, ^57^ ^]^ together with our dataset in sheep. To identify a developmental coordinate of the pluripotency spectrum and explore the correlations of different cell types across species, we integrated the scRNA‐seq data of mouse (E3.5‐E6.5), pig (E5‐E12), sheep (E5‐E12) ICM cells and epiblast cells. By dimensionality reduction analysis, we found that pig and sheep epiblasts at equivalent embryonic days showed a higher correlation than mouse (**Figure** **3**A). Moreover, the cells of same pluripotent state among species could be grouped together, so we supposed that mouse E3.5 ICMs are closer to pig and sheep E5‐E6 ICMs, mouse E4.5 pre‐EPIs resemble pig and sheep E7‐E8 pre‐EPIs, and mouse E5.5 formative EPIs are closer to pig and sheep E9‐E10 EPIs. Similarly, mouse E6.5 primed EPIs correspond to pig and sheep E11‐E12 EPIs.

Next, we analyzed the expression of homologous genes in epiblast development. Our results show that pigs and sheep exhibit more similarities in gene expression pattern and morpholoy of the epiblast with a rapid decline of naïve pluripotent markers and maintain a relatively stable formative pluripotent state during epiblast pluripotent transition when compared to mouse (Figure 3B,C). To establish the culture conditions of sheep PSCs, we focus on analyzing the expression profiles of key genes in JAK‐STAT, WNT, FGF, and TGF‐β signaling pathways. We found that most genes in the JAK‐STAT signaling pathway were highly expressed in a naïve state but down regulated after the epiblast formation, which indicates that the activation of JAK‐STAT is crucial for naïve pluripotency. We also note that the activity of WNT, FGF, and TGF‐β signaling pathways significantly increased during the epiblast from naïve to formative state transition, suggesting that inhibition of WNT signaling and activation of FGF, TGF‐β signaling may be required for the maintenance of stable epiblast stem cells (Figure 3D). Although the signaling pathways related genes expression trends are roughly similar among species, the specific gene expression levels are different. Representative genes related to naïve pluripotency such as *KLF2* exhibited notably higher expression in mouse, but *KLF17* specifically expressed in pig and *TFAP2C* is upregulated in sheep naïve state. In addition, when compared to mouse, the expression of signaling pathway related genes of naïve state including *IL6* showed higher expression in sheep and pig, while *BMP4* almost hardly expressed. Interestingly, we found SRC signaling family member *LCK* highly expressed in sheep formative and primed epiblasts, which may affect the development process of epithelial‐to‐mesenchymal transition (Figure 3E). Based on these findings, we designed culture conditions for sheep stable epiblast pluripotent stem cells.

In summary, our comparative analysis depicted a developmental coordinate of the naïve, formative, and primed pluripotency state of the epiblast lineage among sepcies. Our findings showed an overall conservatism trend of epiblast development in pluripotency transition and signalings in mouse, pig, and sheep. But pig and sheep exhibit more similarities in embryo morphology, gene expression pattern and pluripotent signaling requirements when compared to mouse. Meanwhile, divergences in species‐specific gene expression pattern and regulatory networks were also observed.

### 2.4 Establishment and Characterization of Sheep Formative PSCs (sfPSCs)

We have demonstrated that sheep epiblast pluripotency undergoes three pluripotency states with a rapid loss of naïve pluripotency and a relatively stable formative and primed state. Therefore, we considered whether different pluripotent state PSCs could be established from the embryo epiblast. To optimize the culture medium, we focused on pluripotent signaling pathways of JAK/STAT3, WNT, FGF, and TGF‐β. We found that *IL6* and *IL6R* were highly expressed in ICMs and pre‐EPIs, therefore, the cytokines IL6 and sIL6R were added to the medium. WNT proteins activated intracellular signaling pathways that participate in embryonic development, such as cell fate specification, tissue patterning, and organogenesis.^[^ ^58^ ^]^ We found WNT relative genes begin expression at E8 and are significantly upregulated starting from E10, emphasizing the importance of WNT inhibition. IWR1 and GSK3β co‐inhibition could stabilize β‐catenin in the cytoplasm and promote PSCs homogeneity, so we chose both IWR1 and CHIR99021.^[^ ^59^, ^60^ ^]^ In addition, the receptors of Activin A and FGF2 are highly expressed in sheep epiblasts from E8, which consist with the previous report of the pluripotency of epiblasts require activated FGF and TGF‐β pathways.^[^ ^9^, ^61^ ^]^ Interestingly, we found non‐receptor protein‐tyrosine kinase family member *LCK* expressed in high levels, which could couple with many growth factor receptors to regulate cell survival, proliferation and epithelial‐to‐mesenchymal transition.^[^ ^62^ ^]^ These implicate non‐receptor protein‐tyrosine kinases signaling is essential for sheep PSCs self‐renewal. Hence, we developed a serum‐free N2B27 basal medium with IL6, sIL6R, FGF2, Activin A and three inhibitors of GSK3β, WNT, and LCK. Moreover, to safeguard the viability of cells, we added ascorbic acid and ROCK inhibitor (Y‐27632).

**Figure 4:**
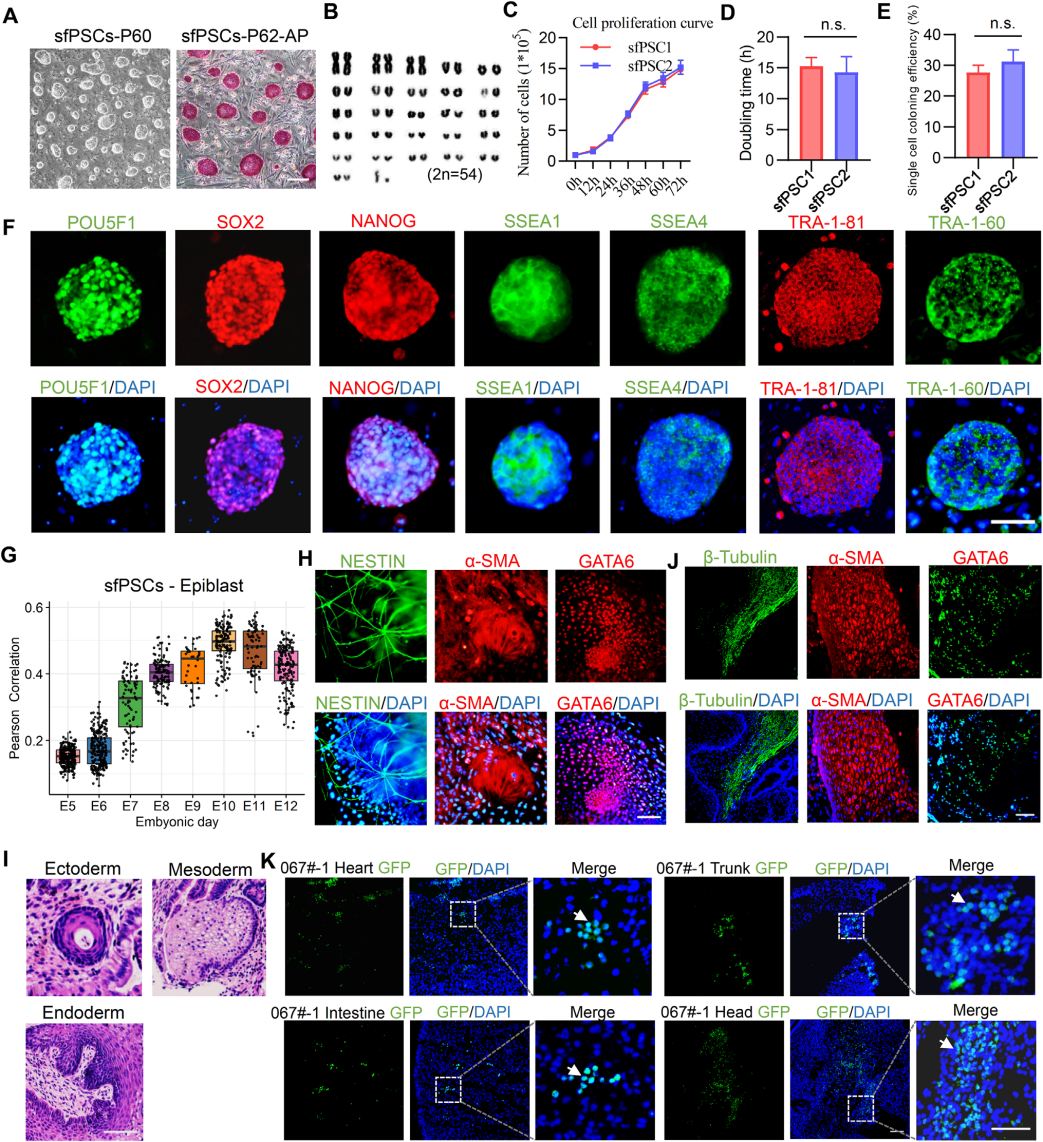
Derivation and characterization of sfPSCs. A) Morphology and alkaline phosphatase staining of sfPSCs cultured on feeders at different passages. Scale bar, 100 µm. B) Karyotype analysis of sfPSCs at passage 50. C) Cell proliferation curve of sfPSC1‐P30 and sfPSC2‐P33. D) Population doubling time of sfPSC1‐P32 and sfPSC2‐P35. E) Single cell cloning efficiency of sfPSC1‐P32 and sfPSC2‐P35. F) Immunostaining of pluripotent markers POU5F1, SOX2, NANOG, SSEA1, SSEA4, TRA‐1‐81, TRA‐1‐60, in sfPSCs, DAPI was used to stain nuclei. Scale bar, 50 µm. G) The box plot displays the Pearson correlation coefficients between epiblasts at different embryonic stage and the sfPSCs transcriptomes. The box in the plot represents the interquartile range (from the lower to the upper quartile), with the horizontal line indicating the median. H) Immunostaining for EBs differentiation assay of three germ layers. The nuclei were stained with DAPI. Scale bar, 50 µm. I) Representative images of teratoma sections haematoxylin and eosin (H&E) staining with ectoderm lineage (neural rosette), mesoderm lineage (cartilage) and endoderm lineage (epithelium). Scale bars, 100 µm. J) Representative immunofluorescence images showing the presence of ectoderm/mesoderm/endoderm in teratomas. Scale bar, 100 µm. K) Detection of sfPSCs GFP signal in the chimera fetus. A higher magnifications are shown at the right and the arrows indicate representative cells that were donor‐cell descendants. The nuclei were stained with DAPI. Scale bars, 200 µm.

Epiblasts were isolated from E8 late blastocysts and E10 bilaminar embryos in vivo. Up to 100% epiblasts formed outgrowth within 4‐5 days. Colonies were mechanically picked for each embryo, accutase dissociated into several clumps, and plated onto new feeder cells for subculture. Finally, six cell lines were established. The morphologies of the colonies are domed with smooth edges (**Figure** **4**A). These cell lines could maintain in long‐term cultures for over 100 passages with a normal karyotype (Figure 4B). They proliferated robustly and routinely passaged every 3 days (1:4 passaging ratio). The doubling time was ≈15 h (Figure 4C,D). The colony formation efficiency was ≈30% (Figure 4E). For pluripotent features, these PSCs exhibited alkaline phosphatase (AP) activity (Figure 4A) and positive for the pluripotency markers, such as POU5F1, SOX2, NANOG, SSEA1, SSEA4, TRA‐1‐60 and TRA‐1‐81 (Figure 4F). At the pluripotency transcriptional level, spearman's correlation analysis further revealed that these PSCs exhibited a stronger correlation with the E10 epiblasts (Figure 4G). Therefore, we provisionally termed them sheep formative pluripotent stem cells (sfPSCs).

To evaluate the in vitro differentiation properties, we found that sfPSCs could form embryonic body (EB) in suspension culture after 4‐5 days. When seeded on plates coated with gelatin for an additional 5‐7 days, EBs spontaneously differentiated into three germ layers, as determined by immunofluorescence positive for NESTIN (ectoderm marker), α‐SMA (mesoderm marker), and GATA6 (endoderm marker) (Figure 4H). RT‐PCR analysis of EBs and EBs differentiated cells showed that the three germ layer marker genes were upregulated (Figure S5A, Supporting Information). To test the in vivo differentiation ability, we injected sfPSCs into nude mice and teratomas were obtained 4‐6 weeks later. Histological analysis showed that the teratomas containing tissues including ectoderm lineage (neural rosette), mesoderm lineage (cartilage) and endoderm lineage (epithelium) (Figure 4I,J), representative differentiation genes also detected by RT‐PCR (Figure S5B, Supporting Information). To test whether sfPSCs can be incorporated into the embryos and form chimeras, we injected GFP‐labeled sfPSCs into E5.5 early blastocysts. GFP‐sfPSCs were detected in chimeric blastocysts (Figure S5C,D, Supporting Information). Immunofluorescence staining and cell number analysis revealed that sfPSCs could connect with the host embryos and maintained proliferate after injection (Figure S5E, Supporting Information). A total of 11 fetus were harvested from 5 pregnant at day 26‐28 of gestation following the transfer of the chimeric blastocysts to synchronized recipient sheep (Table S6, Supporting Information). Frozen section analysis of the chimera fetus tissues showed that despite the low contribution of the donor cells, GFP positive signal were found in multiple tissues and organs (Figure 4K). Genomic DNA PCR assays further demonstrated GFP DNA in those chimeras (Figure S5F, Supporting Information). Collectively, these results confirm that sfPSCs have the ability of contribution to ICM. We then performed cell nuclear transfer assays using GFP‐sfPSCs as nuclear donor cells. Compared with the formation rate of parthenogenetic blastocysts and SEF‐SCNT blastocysts, the results demonstrated that sfPSCs can be used as donor cells for efficiently producing cloned embryos in further applications (Figure S5G and Table S7, Supporting Information).

### 2.5 Defining Factors Required for Long‑Term Maintaining sfPSCs

**Figure 5:**
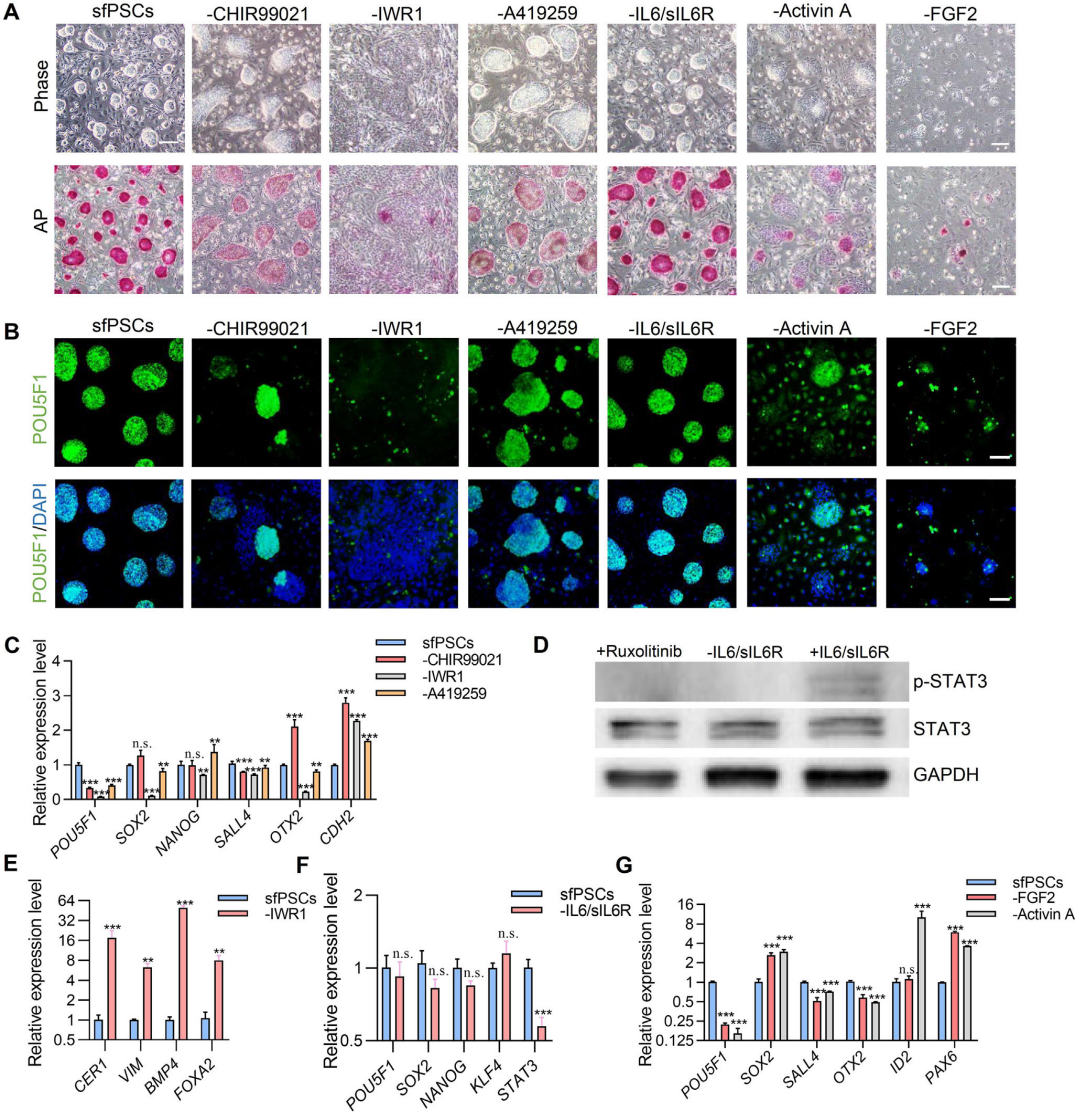
Identification of signaling components required for sfPSCs pluripotency. A) Representative images of the morphology and AP staining in sfPSCs colonies after withdrawal of CHIR99021, IWR1, A419259, IL6/sIL6R, Activin A or FGF2 from the culture medium. Scale bar, 100 µm. B) Immunostaining of pluripotency marker POU5F1 expression by omission of the individual components. The nucleus is indicated by DAPI. Scale bar, 100 µm. C) Quantitative analysis of pluripotent marker genes expression after removal of CHIR99021, IWR1, A419259. D) Western blot analysis of phosphorylated STAT3 (p‐STAT3) in sfPSCs upon IL6/sIL6R withdrawal. E) Quantitative analysis of mesendoderm marker genes expression after removal of IWR1. F) Quantitative analysis of pluripotent marker genes expression following the removal of IL6/sIL6R. G) Quantitative analysis of marker gene expression after removal of FGF2 and Activin A. For C and E–G, the error bar indicates ± SD (*n* = 3, independent experiments).

We removed individual components from the culture medium to investigate the roles of the signaling requirements in sfPSCs. Withdrawal of CHIR99021 resulted in the heterogeneous expression of POU5F1 and weakened the AP staining signal (**Figure** **5**A,B). Removal of IWR1, a tankyrase inhibitor of the canonical WNT/β‐catenin signaling pathway, caused a rapid loss of colony morphology, the appearance of differentiated cells, and significant downregulation of pluripotency factors such as *POU5F1*, *SOX2*, *NANOG*, *SALL4*, and *OTX2* (Figure 5A–C). Meanwhile, the expression of the mesoderm and endoderm differentiation marker *CER1*, *VIM*, *BMP4*, and *FOXA2* upregulated (Figure 5E). The colony flattened when A419259 was removed from the medium (Figure 5A). We further identified the function of growth factors. We found that the withdrawal of IL6/sIL6R did not affect the cell morphology and the pluripotency of sfPSCs, but the expression of JAK/STAT signaling pathway downstream transcription factors STAT3 and the p‐STAT3 protein downregulated (Figure 5A,B,D,F). We observed the removal of either FGF2 or Activin A significantly impaired pluripotency and resulted in gradual differentiation by the loss of *POU5F1* and upregulation of neural markers *PAX6* expression (Figure 5A,B,G). In addition, cell proliferation is severely influenced by attenuating FGF2. SMAD signaling downstream differentiation associated genes were upregulated by omission of Activin A (Figure 5A,G). These results indicated that the defining factors are required for the long‑term maintenance of sfPSCs.

For the analysis of the regulatory targets of WNT signaling pathway, we perform transcriptome sequencing of sfPSCs with removing individual small molecules of WNT signaling pathway (CHIR99021, IWR1, A419459 respectively). PCA analysis showed the separation trends among the global transcriptome expression profiles of the four samples (Figure S6A, Supporting Information). The Venn diagram delineates the sets of genes that are common and specifically expressed in pairwise differential expression analyses (Figure S6B, Supporting Information). Functional enrichment analysis of down regulated DEGs in the group of withdrawal CHIR99021 were enriched in the terms related to cell proliferation, cell growth and cell cycle, suggesting that the cell proliferation require the presence CHIR99021 (Figure S6C, Supporting Information). The up regulated DEGs in the group of withdrawal A419259 were enriched in the terms of epithelial cell proliferation and epithelial to mesenchymal transition, indicating the existence of the EMT process (Figure S6D, Supporting Information). The up regulated DEGs in the group of withdrawal IWR1 were enriched in the terms related to morphogenesis, cell fate commitment, and endoderm‐mesoderm development, suggesting that the PSCs undergoes differentiation (Figure S6E, Supporting Information). In addition, by removing of IWR1 resulted in a significant downregulation of pluripotency genes such as *LIN28B*, *EZH2*, *PRDM14*, *ZIC2*, *POU5F1*, *SOX2*, *UTF1*, while the expression of mesodermal and endodermal differentiate gene such as *LGR5*, *KRT19*, *GATA4*, *DAB2*, *HNF4A*, *FOXA1*, *EOMES*, *SOX17* prominent upregulation. Moreover, we found that WNT ligand genes *WNT11*, *WNT9A*, and *WNT2B*, receptor genes *LRP5*, as well as other regulatory factor *DKK1*, *SFRP5* were highly expressed since IWR1 withdrawal (Figure S6F, Supporting Information). Compared to previously reported sheep PSCs,^[^ ^35^, ^36^ ^]^ the sfPSCs enhanced the ability of suppressing genes associated with WNT signaling pathway, which may facilitate the sfPSCs long‐term passage and pluripotency maintenance (Figure S7A–C, Supporting Information). These results suggest that inhibiting the WNT signaling pathway is important to maintain sheep PSCsself‐renewal.

### 2.6 Generation of Sheep Primed PSCs (spPSCs) in WNTi Medium

Primed PSCs are derived from anterior gastrula primitive streak epiblast, the most proximal pluripotent tissue to the early somatic and germ cell precursors.^[^ ^63^ ^]^ Our insights from scRNA‐seq data indicate that during sheep epiblast development, the classical primed pluripotency genes exhibited relatively high expression from E12. We then attempted to generate sheep primed PSCs (spPSCs) from E12‐E14 embryos. Firstly, we focused on identifying culture conditions that support the primed state. We note that FGF and TGF‐β signaling are activated in primed epiblasts (Figure 2E), suggesting that the maintenance of primed pluripotency requires the presence of FGF2 and Activin A. Further analysis showed that E12 epiblasts exist at the critical point of gastrulation, so WNT signaling inhibitors are indispensable for primed PSCs self‐renewal. Finally, we established a condition consisting of Activin A, FGF2, and IWR1 (AFI), which successfully supported spPSCs outgrowth formation from E12‐E14 epiblast with high efficiency.

**Figure 6:**
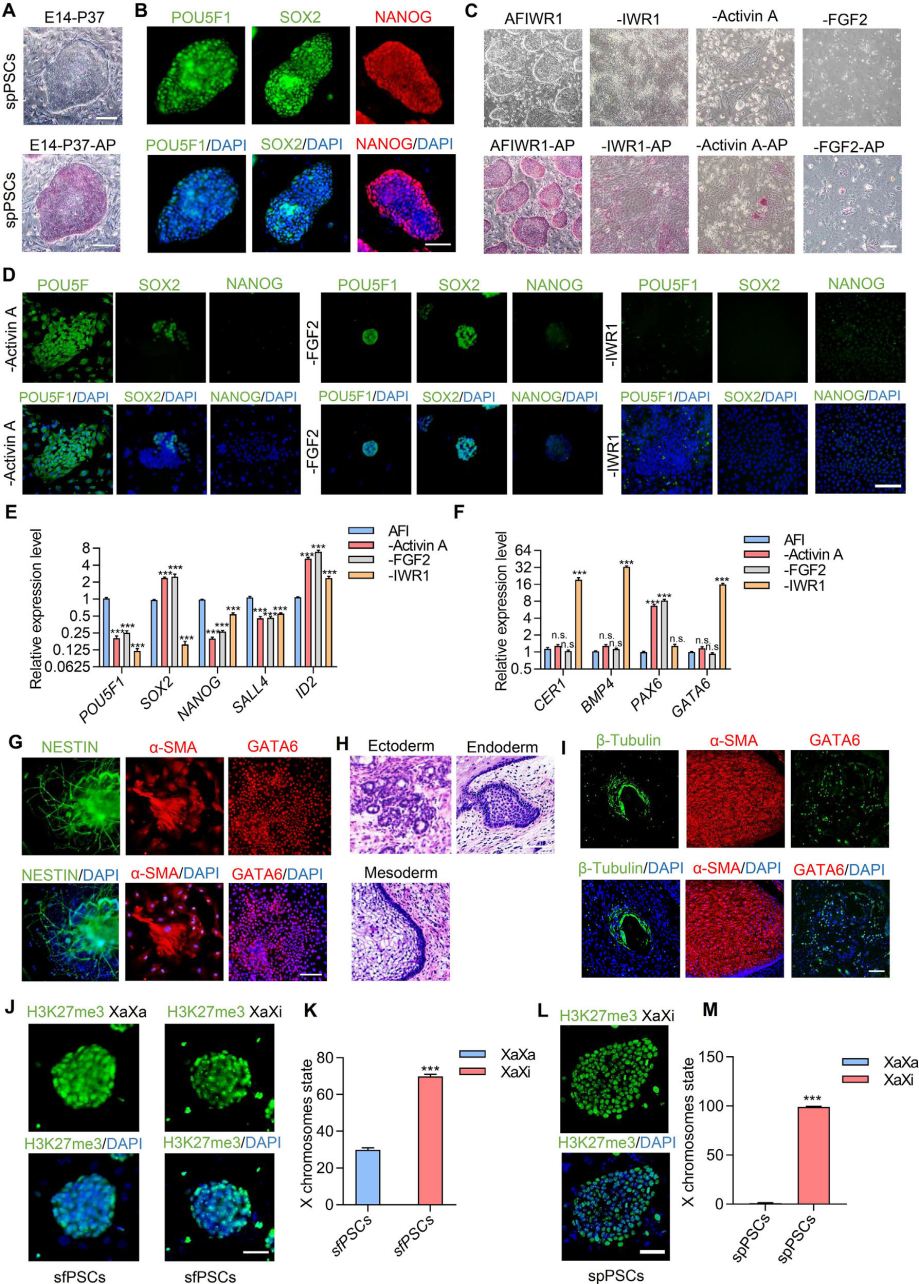
Derivation and characterization of spPSCs. A) Morphology and AP staining of spPSCs. Scale bar, 50 µm. B) Immunostaining of pluripotent markers POU5F1, SOX2, and NANOG of spPSCs in AFIWR1 medium, DAPI was used to stain nuclei. Scale bar, 50 µm. C) Representative images of colony morphology and AP staining in spPSCs after removal of IWR1, Activin A or FGF2 from the AFI medium. Scale bar, 100 µm. D) Immunostaining of pluripotency marker POU5F1, SOX2, NANOG expression by omission of the individual components. The nucleus is indicated by DAPI. Scale bar, 100 µm. E) Quantitative analysis of pluripotent marker genes expression after removal of individual factors. F) Quantitative analysis of differentiation marker genes expression after removal of individual factors. G) Immunostaining for EBs differentiation assay of spPSCs. The nuclei were stained with DAPI. Scale bar, 50 µm. H) Representative images of teratomas stained with H&E, displaying ectoderm, mesoderm and endoderm lineage. Scale bars, 100 µm. I) Immunofluorescence images showing the presence of three germ layer cells in teratomas derived from spPSCs. Scale bar, 100 µm. J) Immunostaining detection of H3K27me3 foci in female sfPSCs. Scale bar, 100 µm. K) The ratio of XaXi and XaXa in female sfPSCs. L) Immunostaining detection of H3K27me3 foci in female spPSCs. Scale bar, 100 µm. M) The ratio of XaXi and XaXa in female spPSCs. For E, F, and K, M, the error bar indicates ± SD (*n* = 3, independent experiments).

We established stable cell lines from E12 and E14 embryos respectively. The colonies of spPSCs were flattened (**Figure** **6**A), similar to those of primed PSCs, as represented by mouse EpiSCs and human ESCs.^[^ ^9^, ^61^ ^]^ These cells exhibited AP activity (Figure 6A) and could passage by accutase every four days for long‐term culture (over 100 passages) with a normal karyotype. Immunostaining showed that spPSCs expressed the pluripotency markers *POU5F1*, *SOX2*, and *NANOG* (Figure 6B). To identify the pathway necessary to sustain undifferentiated spPSCs, we tested the requirements of each factor in the culture medium. We found that the removal of IWR1 disrupted the boundaries of typical clonal morphology after passages and significant downregulation of pluripotency factors and upragulation of differentiation genes such as AP, *POU5F1*, *SOX2*, *NANOG*, *CER1*, and *GATA6* (Figure 6C–F). We next tested whether IWR1 could be replaced with other WNT inhibitors, and we found that the functions of IWR1 can mimic by IWP2, which blocks WNT secretion and abrogates WNT signaling from the endogenous sources of WNT, and XAV939 which can attenuate the tankyrase activity and interrupt WNT signaling mediated by *CTNNB* (Figure S8A–F, Supporting Information).^[^ ^64^, ^65^, ^66^ ^]^ In addition, we found that when FGF2 and Activin A were withdrawn, spPSCs could not proliferate well, and colonies became differentiated (Figure 6C–F). These results suggest that AFWNTi (Activin A, FGF2 and WNT inhibitor) medium is minimal requirements for the generation of spPSCs. To test the differentiation potential of spPSCs, results indicated that spPSCs could form EBs in vitro and spontaneously differentiate into three germ layer cells, as determined by the immunofluorescence assay (Figure 6G). When the spPSCs were subcutaneously injected into nude mice, teratomas were obtained in vivo, and histological examination revealed that the teratomas contained tissues of all three germ layers (Figure 6H,I).

Compared with sfPSCs in gene expression, the classic pluripotent markers *POU5F1*, *SOX2*, S*ALL4*, and the formative pluripotent marker *ZIC3*, *OTX2* were downregulated in spPSCs. Conversely, the primed and epithelial‐mesenchymal transition marker *VIM*, *EOMES*, *BMP4*, *TCF7L2*, *CDH2* were significantly upregulated (Figure S8E,F, Supporting Information). By immunostaining of β‐catenin protein, we further demonstrated that β‐catenin stabilizes the pluripotency of sfPSCs and spPSCs through high expression in the cytoplasm (Figure S8I, Supporting Information). We also performed X chromosome status analysis of female sfPSCs and spPSCs. The data revealed a predominance of completely inactivated X chromosomes (>70%) coexisting with a minor population retaining actived X chromosomes (<30%) in sfPSCs. While, spPSCs are almost 100% in inactivated state (XaXi). This mixed X chromosome activation state could demonstrates that sfPSCs and spPSCs exhibit different epigenetic features of formative and primed pluripotency (Figure 6J–M).

### 2.7 Transcriptional Features and Chromatin Accessibility in sfPSCs and spPSCs

**Figure 7:**
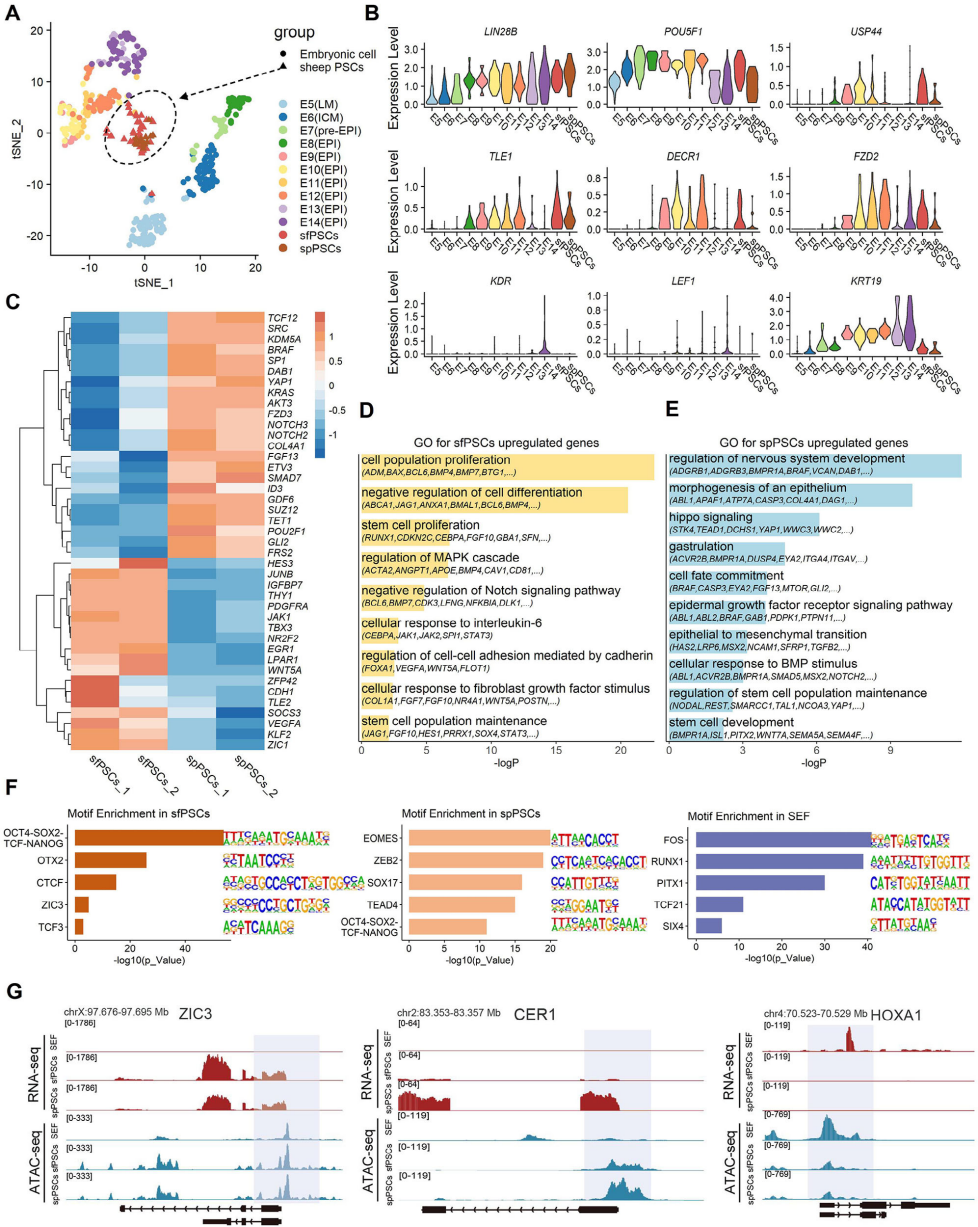
Molecular features of sfPSCs, spPSCs, and SEFs based on RNA‐seq and ATAC‐seq analyses. A) The t‐SNE plot depicts the distribution of scRNA‐seq data obtained from sheep epiblast cells (E5‐E14) and PSCs, with shapes representing different groups and colors representing different cell stages. B) The violin plot displays the expression levels of pluripotency and differentiation genes in sheep EPI lineage as well as different pluripotent state of PSCs. C) Heatmap showing the differentially expressed genes between the sfPSCs and spPSCs transcriptomes, along with unsupervised hierarchical clusters of the samples. D) GO enrichment terms of differentially expressed genes upregulated in sfPSCs. E) GO enrichment terms of differentially expressed genes upregulated in spPSCs. F) Transcription factor binding motifs enriched in specific open chromatin regions in sfPSCs, spPSCs, and SEF. G) ATAC‐seq and RNA‐seq data tracks for the vicinity of transcription factors ZIC3, CER1, and HOXA1.

To further investigate the differences between sfPSCs and spPSCs at the molecular level, we compared the single‐cell transcriptomes of sfPSCs and spPSCs from different embryonic stages to those of sheep epiblast lineages from E5 to E14. t‐SNE visualization showed that sheep PSCs were clustered in an independent group. (**Figure** **7**A). Violin plots showing the expression levels of pluripotent genes of *POU5F1*, *LIN28B* were highly expressed, but WNT pathways and gastrulation relative genes of *LEF1*, *KDR*, and *KRT19* were barely expressed in these two cell types (Figure 7B), which suggests that sfPSCs and spPSCs maintain the pluripotency of epiblast cells. Compared to spPSCs, sfPSCs exhibited higher expression levels of pluripotent markers *USP44*, *TLE1*, *DECR1*, and *FZD2*, which were associated with formative pluripotency (Figure 7B). DEGs analysis by RNA‐seq data further indicated that the expression of classical formative markers (e.g., *ZIC1*, *CDH1*, *ZFP42*, *IGFBP7*, *EGR1*, *TLE2*) was upregulated in sfPSCs, and the expression levels of primed related genes (e.g., *GLI2*, *ETV3*, *ID3*, *NOTCH2*, *SRC*, *DAB1*, *BRAF*, *YAP1*) were higher in spPSCs (Figure 7C; Table S5, Supporting Information). Functional enrichment analyses demonstrated that upregulated DEGs in sfPSCs were enriched in terms such as cell proliferation, negative regulation of Notch signaling pathway, response to interleukin‐6, and negative regulation of cell differentiation (Figure 7D). Compared with sfPSCs, DEGs upregulated in spPSCs were involved in terms such as Hippo signaling, epithelial to mesenchymal transition, gastrulation, cell fate commitment and stem cell development (Figure 7E). These results suggest the distinctive transcriptome properties of sfPSCs and spPSCs.

To explore the differences in local accessibility among the genomes of pluripotent cells and terminal lineage cells, we used assay for transposase accessible chromatin (ATAC‐seq) in sfPSCs, spPSCs and sheep embryonic fibroblasts (SEFs) (Figure S9A, Supporting Information). Pearson correlation indicated that the cell types of PSCs and SEFs were clustered separately (Figure S9B, Supporting Information). Although there was a strong correlation between spPSCs and sfPSCs, the signals of the transcription start sites (TSS) to the transcription end sites (TES) are different (Figure S9D, Supporting Information). The distribution of the open chromatin region annotations revealed that ≈15.72% of peaks were enriched in the primarily promoter (≤ 1 kb) region of all samples (Figure S9C, Supporting Information). Next, we analyzed the ATAC‐seq specific peaks (Figure S9E, Supporting Information) and the enrichment of transcriptional factor binding motifs of each cell type by pairwise comparison. As expected, OCT4‐SOX2‐TCF‐NANOG complex binding motifs that were important in maintaining the pluripotency of PSCs were enriched in both sfPSCs and spPSCs. In sfPSCs, the binding motifs of transcriptional factors, including CTCF, OTX2, ZIC3, and TCF3 were abundant. These factors were significant for formative pluripotency maintenance. For spPSCs, the binding motifs of transcriptional factors EMOES, ZEB2, SOX17, and TEAD4 were enriched, which could regulate the primed pluripotent state and further affect the cell fate determination. We also found the transcriptional factors of FOS, RUNX1, PITX1, and TCF21 were highly enriched in SEFs, which were associated with cell differentiation (Figure 7F). Then, we used integrative genomics viewer (IGV) snapshot to display the open chromatin regions of pluripotent genes and their transcriptional features. In contrast with SEFs, we found strong ATAC‐seq peaks of *ZIC3*, *OTX2*, and *SOX2* at the promoter of sheep PSCs and *CER1* is special enrichment in spPSCs. Meanwhile, RNA‐seq reads of pluripotent genes were highly enriched in sfPSCs and spPSCs but rarely detected in SEFs. Instead, the specific peaks of differentiation gene *HOXA1* and *VIM* were strongly enriched in SEFs (Figure 7G; Figure S9F, Supporting Information). According to ATAC‐seq results, we found the distinctions in chromatin accessibility between sheep PSCs and SEFs. Moreover, the diverse enrichment of transcriptional factor binding motifs and the different IGV peaks involved in pluripotency indicated the different pluripotent state of sfPSCs and spPSCs.

## 3 Discussion

Our research provides a comprehensive scRNA‐seq profile of early sheep embryonic development. We further explore the lineage segregation process, the molecular features of epiblast pluripotency changes, and the cross‐species comparison. We found that although embryo development shows broad conservativeness between mammals, pigs and sheep exhibit more similarities in embryo morphology, gene expression pattern of the epiblast and pluripotent signaling requirements in early embryonic development when compared to mice. For example, mouse embryo with the formation of the cup‐shaped post‐implantation epiblast while pig and sheep embryos undergo a protracted developmental period with the formation of a flat bilaminar embryonic disc.^[^ ^18^ ^]^ In addition, sheep and pig embryo naïve pluripotent markers decline sharply during the lineage differentiation, and the formative pluripotent state can maintain a relatively stable long time in pregastrulation epiblasts. The specific gene expression levels and regulation signalings are still different among species. For instance, LIF is essential for maintaining mouse naïve pluripotency but seems ineffective for pig and sheep naïve states.^[^ ^18^, ^67^ ^]^ WNT inhibitor is indispensable for pig, bovine, sheep formative and primed pluripotency but dispensable for mouse and human.^[^ ^9^, ^11^, ^23^, ^24^, ^36^ ^]^ Based on scRNA‐seq analysis, we defined signaling molecules required for establishing and maintaining pluripotency of sheep epiblast stem cells. Stabled formative and primed PSCs efficiently generated from sheep pre‐gastrulation epiblasts. These cells exhibited long‐term passage ability, maintained normal karyotype, expressed pluripotent marker genes and possessed the differentiation capacity for three germ layers. Notably, sfPSCs and spPSCs displayed different pluripotency gene expression patterns and epigenetics status corresponding to the formative and primed state of embryo epiblast respectively.

Naïve, formative and primed state PSCs with distinct molecular features and developmental potential.^[^ ^4^, ^5^ ^]^ Various combinations of cytokines, growth factors have been explored to facilitate the maintenance of PSCs cultured in vitro.^[^ ^68^, ^69^ ^]^ PSCs with different pluripotent states in mice, humans, and monkeys have been established and studied extensively. It is also critical to develop efficient and stable culture conditions for defined pluripotent state PSCs of large animals for their utilization in disease modeling and basic studies.^[^ ^28^ ^]^ Current results suggest that WNT signaling inhibition is required for the derivation of livestock PSCs.^[^ ^23^, ^33^, ^70^ ^]^ Nonetheless, not all WNT inhibitors are effective in different species; the interaction between various components in the medium and the relationship between different pluripotency factors are not yet clearly elucidated.^[^ ^56^ ^]^ The regulatory pathway and underlying targets of IWR1, XAV939, and IWP2 in spPSCs also remain to be determined.

Mouse, rat, and monkey naïve PSCs can contribute to germline chimeras, whereas no livestock PSCs with germline transmission ability are available.^[^ ^71^ ^]^ Previous studies indicated that mouse formative PSCs are competent for germline chimera formation.^[^ ^10^, ^11^, ^72^ ^]^ Given that, we also tested the chimera development potential of sfPSCs. However, the present evidence showed that sfPSCs exhibit a lower degree of chimerism in the fetus tissues after blastocyst injection. The reasons maybe the pluripotent state of sfPSCs that are incompatible with the injected embryo development stage. In this study, we successfully established stable sfPSCs and spPSCs while failing to establish sheep naïve PSCs. The naïve culture conditions applied in mice and humans do not work in sheep, and it becomes evident that the reliance on signaling pathways of naïve pluripotency varies across species. However, it should be noted that the activation of JAK/STAT3 and inhibition of FGF signaling seems to be a universal feature for naïve PSCs among species, but the balance of JAK/STAT3 and FGF signaling should be further discussed, and the exact mechanisms are unclear. In future studies, it will be essential to optimize naïve culture conditions and test whether sheep naïve PSCs can generate a higher degree of chimerism.

Large‐scale single‐cell sequencing methods help completely dissect the complicated pluripotency regulation networks to derive PSCs and provide a reference for evaluating PSCs pluripotency. Furthermore, the cross‐species comparison of single‐cell transcriptome data will serve as a resource for advancing of our knowledge of the conservation and divergence of mammalian embryo development. Establishment of sfPSCs and spPSCs will be beneficial for exploration of self‐renewal mechanisms of PSCs among species, and the combination of stable livestock PSCs with multiple gene editing alongside somatic cell nuclear transfer techniques will hold promising potential for applications in human disease modeling, regenerative medicine and stem cell‐based breeding in agriculture.

## 4 Experimental Section

### Animal Treatment and Ethics Statement

All mouse and sheep procedures conducted in the laboratory were approved in advance by the Institutional Animal Care and Use Committee (IACUC) of China Agricultural University (Approval Number:AW40904202‐3‐1) and the IACUC of Beijing University of Agriculture (Approval Number:BUA612412117). CD‐1® (ICR) IGS mice and BALB/c nude mice were purchased from Beijing Vital River Laboratory Animal Technology Co., Ltd, which were utilized for the isolation of mouse embryonic fibroblasts (MEFs) and teratoma formation experiments. All sheep were in natural estrus and were mated for the purpose of collecting embryonic single cells and deriving pluripotent stem cells.

### Collection of Sheep Embryos from E1‐E14 and Isolation of Embryonic Single Cells

Sheep embryos at embryonic days E1‐E14 were collected by flushing the fallopian tubes or uterine horns with embryo washing buffer (DPBS + 2% FBS) and transported to the laboratory in a portable incubator maintained at 37 °C. The zona pellucidae were removed using pronase, and the embryos were then transferred to the washing buffer (DPBS + 0.1% BSA) for further cleaning. Blastomeres from E1‐E4 embryos were mechanically dissociated using a pulled glass capillary and collected into lysis buffer. For E5‐E14 embryos, single‐cell dissociation was performed by incubation in TrypLE™ Express (Gibco, 12605010) at 37 °C for 1‐3 min. The ICMs, epiblasts, hypoblasts, and trophoblast cells were mechanically separated and then subjected to digestion. Subsequently, mRNA reverse transcription and cDNA amplification were performed on the lysed cells.

### Derivation and Culture of Sheep Formative Pluripotent Stem Cells (sfPSCs)

The N2B27 basal medium (500 mL) was prepared as described: 227 mL DMEM/F12 (Thermo Fisher Scientific, 10565‐018), 227 mL Neurobasal (Gibco, 21103‐049), 2.5 mL N2 supplement (Gibco, 17502‐048), 5 mL B27 supplement (Gibco, 12587‐010), 2.5 mL 100× GlutaMAX (Gibco, 35050‐061), 5 mL 100× nonessential amino acids (Gibco, 11140‐050), 0.1 mm β‐mercaptoethanol (Gibco, 21985‐023), 5 mL 100× penicillin‐streptomycin (Gibco, 15140‐122), 5% knockout serum replacement (KOSR, Gibco, A3181502), 50 µg mL^−1^ ascorbic acid (Sigma, A4544) and 2.5 µm ROCK inhibitor Y27632 (Selleckchem, S1049), Small molecules and cytokines were added at the following concentrations: CHIR99021 (1 µm, Selleckchem, S1263), IWR1 (5 µm, Selleckchem, S7086), A419259 (0.3 µm, MedChemExpress, HY‐15764A) or WH‐4‐023 (1 µm, Selleckchem, S7565), recombinant human IL6 (10 ng mL^−1^, PeproTech, AF200‐06), Recombinant human sIL6R (10 ng mL^−1^, PeproTech, 200‐06RC), recombinant human Activin A (25 ng mL^−1^, PeproTech, 120‐14E) and recombinant human FGF2 (12.5 ng mL^−1^, PeproTech, 100‐18B).

For the derivation of sfPSCs from E8 blastocysts, ICMs were isolated and seeded on four‐well plates coated with feeder cells. For the derivation of sfPSCs from E10 epiblasts, the hypoblast and trophoblast cells were mechanically removed, and the epiblasts were dissociated with TrypLE™ Express (Gibco, 12605010) for 3 min and dispersed into small cell clumps before being seeded on four‐well plates coated with feeders. Until initial outgrowths appeared, the outgrowths were mechanically isolated using a mouth pipette and reseeded onto fresh feeders in the medium. sfPSCs were maintained on feeder layers and enzymatically passaged every 3‐4 days at a ratio of 1:3 to 1:5. The process began with a brief wash with DPBS, followed by treatment with Accutase cell dissociation reagent (Gibco, A11105‐01) for 3–5 min. The cells were dissociated and then centrifuged at 1000 rpm for 5 min. After removing the supernatant, sfPSCs were resuspended and seeded in a culture medium under 20% O_2_ and 5% CO_2_ at 37 °C.

### Derivation and Culture of Sheep Primed Pluripotent Stem Cells (spPSCs)

For culturing of spPSCs, N2B27 basal medium supplemented with small molecules and cytokines was used, including recombinant human Activin A (25 ng mL^−1^, PeproTech, 120‐14E), recombinant human FGF2 (12.5 ng mL^−1^, PeproTech, 100‐18B) and IWR1 (5 µm, Selleckchem, S7086). IWR1 can be substituted with XAV939 (5 µm, Selleckchem, S1180) or IWP2 (5 µm, Selleckchem, S7085). For the derivation of spPSCs, E12‐E14 embryonic epiblasts were mechanically isolated and dissociated with TrypLE™ Express (Gibco, 12605010) for 3 min. The cell masses were seeded onto feeders and cultured until outgrowths appeared. Subsequent passages were achieved by mechanically picking the colonies followed by enzymatic digestion.

### Alkaline Phosphatase (AP) Staining

Sheep PSCs were washed with DPBS and fixed in 4% paraformaldehyde (Sangon Biotech, 3053589‐4) at room temperature for 3 min. The fixed cells were washed with DPBS and incubated in AP staining solution (Millipore, SCR004) in the dark at 37 °C for 10 min. Then, the AP staining solution was removed and replaced with DPBS. The stained cells were subsequently observed under the microscope and images were captured.

### Analysis of Cell Growth, Doubling Time, and Single Cell Cloning Efficiency

A total of 1×10^5^ PSCs were seeded per well in 12‐well plates with triplicate repetitions. The cells were digested to single cells and counted by a Luna™ Automated Cell Counter every 12h. Each time point was averaged three times and plotted the cell proliferation curve. The cell doubling time was calculated as previously described.^[^ ^23^ ^]^ For the analysis of single cell cloning efficiency, cells were dissociated into single cells and plated at densities of 100, 200, and 500 cells per well in 6‐well plates in triplicate. Approximately one week later, the colonies were stained AP and the positive colonies were counted for further calculation.

### Karyotyping

Sheep PSCs were cultured to reach 70%–80% confluence and then incubated at 37 °C in fresh medium containing 10% KaryoMAX Colcemid Solution (Gibco, 15210‐040) for 2 h. The cells were subsequently digested into single cells by Accutase, washed with DPBS, and collected by centrifugation. The cell pellets were resuspended in 10 mL pre‐warmed 0.075 M KCL solution (added drop by drop) and incubated at 37 °C for 30 min. Then, 1 mL of pre‐cooled fresh fixative solution (a mixture of methanol and glacial acetic acid in a 3:1 ratio) was added dropwise to the KCL solution and the cells were centrifuged at 1000 rpm for 10 min at 4 °C. The supernatant was discarded and the cell pellet was resuspended in 10 mL of fixed solution incubated on ice for 30 min. The fixation procedure was then repeated with fixed solution incubation on‐ice for 1 h. After the final fixation, the cells were resuspended in 300 µL of the fixative solution and dropped onto a precooled slide. After drying thoroughly, the slides were stained with the Rapid Giemsa Staining kit (BBI Life Science, E6073141) and photographed to visualize the chromosomes.

### Immunofluorescence Staining

Sheep PSCs were fixed with 4% paraformaldehyde at room temperature for 30 min and washed with DPBS. Then, the cells permeabilized in 0.5% Triton X‐100 for 20 min and blocked with 3% BSA (Sigma, A1470) for 1h. Primary antibodies were incubated at 4 °C overnight. The cells were washed with DPBS for 5 min three times. Secondary antibodies conjugated to Alexa Fluor (Invitrogen) were incubated at room temperature for 1 h and then washed with DPBS three times. For nuclear staining, the cells stained with DAPI (Roche Life Science, 10236276001) for 3  min and imaged by a fluorescence microscope. The antibodies used are listed in Table S8 (Supporting Information).

### Embryoid Body Differentiation

Sheep PSCs were dissociated into single cells by accutase and seeded in 35‐mm low‐attachment plates for 5‐7 days with differentiation medium on a horizontal shaker at 70 rpm. The differentiation medium consisted of DMEM (Gibco, 11960‐044) supplemented with 10% FBS (Gibco, 16000‐044), 1% GlutaMAX (Gibco, 35050‐061), 1% penicillin‐streptomycin (Gibco, 15140‐122). For further differentiation, embryoid bodies were selected and plated on 0.1% (w/v) gelatine‐coated 12‐well plates for another 5‐7 days in the same differentiation medium. The medium changed every two days. After the differentiation period, the cells were fixed and immunofluorescence was used to evaluate the differentiation into the three germ layers.

### Teratoma Formation and H&E Staining

Approximately 1×10^7^ Sheep PSCs were suspended in 150 µL culture medium and subsequently injected into BALB/c nude mice. After 4–6 weeks post‐injection, teratomas were harvested from the subcutaneous layer of the mice. The collected teratomas were washed twice in DPBS and fixed with 4% PFA for 2 days at 4 °C. The tissues were dehydrated with an alcohol gradient (70%, 80%, 90%, 95%, and 100% for 1 h each), transferred into xylene and embedded in paraffin. Samples were cut into 5 µm thickness, deparaffinized in xylene and rehydrated with decreasing concentrations of ethanol. Tissue slice were then stained with haematoxylin (Sigma, MHS16) and eosin (Sigma, HT110116) to assess the differentiation of various cell types within the teratoma.

### RT‐qPCR Analysis

Total RNA were extracted using the RNA prep Pure Cell/ Bacteria Kit (TIANGEN, DP430) according to the manufacturer's protocol. Complementary DNA (cDNA) was prepared using the 5 × All‐In‐One RT Master Mix (Abm, G490). RT‐qPCR reactions were performed using 2 × RealStar Green Power Mixture (GenStar, A311‐05) on a LightCycler 480 II Real Time System (Roche). Gene expression levels was calculated relative to GAPDH using the comparative CT (2^‐ΔΔCT^) method. Three biological replicates were conducted for all experiments. The primer sequences information are listed Table S9 (Supporting Information).

### Western Blotting

Total protein were extracted by cell lysis buffer (Beyotime Biotechnology, P0013) supplemented with protease inhibitor cocktail to prevent degradation (Beyotime, Cat # P1050). The concentrations of the extracted proteins were quantified by the BCA protein assay kit (Sangon Biotech, C503051). Then, 15 µg of protein from each sample was electrophoresed by 8% sodium dodecyl sulfate polyacrylamide gel electrophoresis and then transferred to polyvinylidene fluoride membranes. The blots were blocked in 5% nonfat powdered milk (Sangon Biotech, A600669‐0250) at room temperature for 1 h and incubated with primary antibodies at 4 °C overnight. Next day, the membranes were rinsed three times for 5 min, followed by incubation in horseradish peroxidase (HRP)‐conjugated secondary antibodies for 1 h at room temperature, and finally rinsed three times for 5 min. The bands were exposed to an enhanced chemiluminescence solution (Gibco, 34075) and analyzed with CLINX chemiluminescence software for quantification.

### Chimera Assay

Sheep early blastocysts of E5.5 were collected in embryo flushing solution from the uterus. The embryos were transferred into optimized TCM‐HEPES medium composed of 2 mm NaHCO_3_ (Sigma, S5761), 2 mm Sodium pyruvate (Gibco, 11360070), 13.75 mM HEPES‐Sodium (Gibco, 15630080), 1 mm L‐glutamine (Gibco, A2916801), 10% M199 medium (Sigma, M0650), 90% H_2_O (Sigma, W1503) and cultured for ≈0.5 h before injection. For micromanipulation, sfPSCs were digestion and suspended in optimized TCM‐HEPES medium. A XYRCOS‐driven micromanipulator (HAMILTON THORNE, INC. Beverly MA 01915 USA) was used to punch the zona pellucida and trophectoderm under the microscope, 25 sfPSCs were injected into the blastocyst cavities near the inner cell mass. After injection, the embryos were incubate in sfPSCs culture medium for ≈4 h. Then the chimeric embryos were transferred into the uteri of pseudopregnant recipient female sheep.

### Generation of Sheep SCNT Embryos

Sheep ovaries were collected from local slaughterhouse and transported to the laboratory within 2 h in saline supplemented with 100 IU mL^−1^ penicillin and 50 µg mL^−1^ streptomycin. Oocyte complexes were obtained and then cultured in a saturated humidity incubator at 38.5 °C with 5% CO₂ for 19 h. Cumulus cells were removed by digestion with 0.1% hyaluronidase. Matured oocytes in metaphase II were enucleated by micromanipulation and sfPSC was injected into the perivitelline space. The embryos were equilibrated in an incubator for 20 min before being subjected to electrofusion under the following conditions: 1300 V cm^−1^, 25 µs, 1‐s interval, and double pulses. The fused embryos were activated using 5 µm ionomycin (Sigma, I3909) for 5 min, followed by 2 mm 6‐DMAP (Sigma, D2629) treatment for 4 h. Then, the activated embryos were cultured in IVC medium (BOIVC2404).

### Single‐Cell RNA‐seq Processing

For single‐cell RNA‐seq data, umi_tools^[^ ^73^ ^]^ was used to demultiplex the data based on the 8‐bp cell barcodes and 8‐bp unique molecular identifiers (UMIs) in the Read 2 file. The umi_tools extract command was configured with the following parameters: –bc‐pattern = CCCCCCCCNNNNNNNN and –whitelist barcode_list. The barcode sequences and related information for each cell are provided in Table S1 (Supporting Information). The extracted 8‐bp UMIs from Read 2 were subsequently appended to the header line of the paired Read 1 file. For alignment and quantification, the cDNA reference and GTF annotation from the Ensembl Ovis aries Rambouillet dataset (version 110) was used, downloaded from the Ensembl FTP site https://ftp.ensembl.org/pub/release‐110/. Alignment and UMI counting were performed using kallisto (v0.46.0).^[^ ^74^ ^]^

### scRNA‐seq Data Analysis

For the analysis of scRNA‐seq data, R software (version 4.3, www.r‐project.org) was used and Seurat (version 4.1.0.9)^[^ ^75^ ^]^ to load the raw count matrix along with associated metadata. Cell types were defined based on lineage marker genes and the embryonic day of sample collection. To ensure high‐quality data, a series of filtering criteria to the expression matrix was applied: 1) Cells were retained if they expressed more than 2,000 genes.; 2) Cells with a mitochondrial gene UMI proportion exceeding 20% were excluded; 3) Genes detected in fewer than 5 cells were removed from the analysis. After filtering, a total of 773 cells were retained for subsequent analysis (Table S1, Supporting Information). Gene expression was normalized using the LogNormalize method, and the AverageExpression function was applied to calculate the average gene expression for each cell type.

### Gene Co‐Expression Network Analysis

To investigate the gene co‐expression networks during ZGA and lineage differentiation, the WGCNA R package (version 1.68) was employed. First, genes with normalized expression levels below 0.1 in all cells were filtered out. The blockwiseModules function was then used to construct co‐expression modules from the filtered expression matrix. Based on the module eigengenes, signature genes were identified that are significantly associated with specific cell types.

### Gene Module Score Analysis

The AddModuleScore function from the Seurat R package was used to evaluate the expression level changes of different gene modules in the single‐cell dataset. The gene sets for oxidative phosphorylation and glycolysis were constructed based on Gene Ontology entries and relevant metabolic markers.^[^ ^54^ ^]^ The metabolic module scores were visualized using box plots generated with the ggplot2 R package.

### Gene Functional Enrichment Analysis

Functional enrichment analyses of selected genes were performed using Metascape.^[^ ^76^ ^]^ Sheep genes were mapped to their human orthologs, with Homo sapiens as the target species for analysis. Enrichment analyses were conducted using Gene Ontology (GO) biological processes (GO‐BP) and Kyoto Encyclopedia of Genes and Genomes (KEGG) pathways or Wiki pathways. Terms with a minimum count of ≥ 3, an adjusted P‐value < 0.01, and an enrichment factor ≥ 1.5 were considered significant, and similar terms were grouped into clusters. Key pathways were visualized using bar plots in R.

### Cross‐Species Analysis with Public scRNA‐seq Datasets

To perform cross‐species comparisons, epiblast scRNA‐seq data from mouse was integrated, sheep and pig. One‐to‐one orthologous genes were identified using the Ensembl Genome Browser (https://dec2021.archive.ensembl.org) and were used for downstream integration and comparative analysis. Based on the top 2000 highly variable genes in the epiblast lineage, integration anchors were identified using the FindIntegrationAnchors function. The datasets were subsequently integrated using the IntegrateData function based on the these anchors.

### Identification of Differentially Expressed Genes Across Embryonic Stages

Differential gene expression analysis was performed on the TE and epiblast lineages across embryonic stages. The FindMarkers function with the Wilcoxon rank‐sum test was used to compare gene expression between stages. Genes with an absolute average log fold change > 0.25 and a p‐value < 0.05 were considered differentially expressed and used for downstream functional enrichment analysis.

### RNA‐seq Data Processing and Analysis

RNA‐seq data were processed to quantify the expression of protein‐coding genes using Kallisto (version 0.46.0), yielding both estimated read counts and transcript abundance quantified as transcripts per million (TPM). Differentially expressed genes (DEGs) between different cell lines were identified using the DESeq2 tool (version 1.30.1).^[^ ^77^ ^]^ Statistical significance was determined using Benjamini‐Hochberg adjusted false discovery rate (FDR) < 0.05 and an absolute log2 (fold change) > 1 as cut‐off thresholds.

### Transcriptome Correlation Analysis of sfPSCs to Embryonic Cells

The correlation coefficients between sfPSCs and the epiblasts across different embryonic stages were calculated by computing the Pearson's correlation coefficient of the top 2000 variable genes between the TPM values of sfPSCs and single‐cell RNA‐seq data of the epiblasts.

### ATAC‐seq Library Preparation and Data Analysis

ATAC‐seq libraries were prepared following the manufacturer's protocol (Novoprotein, N248). Briefly, 50,000 cells were resuspended in cold lysis buffer and incubated for 5 min. For each sample, 2 µL Tn5 transposase was used to perform tagmentation at 37 °C for 30 min. Subsequently, PCR amplification was conducted using 25 µL of NEBNext High‐Fidelity 2 × PCR Master Mix per sample. Libraries were sequenced with 150‐bp paired‐end reads on the NovaSeq platform. All paired‐end reads were first subjected to adaptor trimming using Trimmomatic (v0.39). The clipped reads were aligned to the sheep genome (Ovis aries ARS‐UI_rAMB_V2, ensembl). Only high‐quality paired reads with a MAPQ score ≥ 30 were retained for further analysis. Peaks were called for each sample using MACS2 (v2.2.7.1). For downstream, we used IDR (2.0.4.2) to identify consistent peaks across three technical replicates and merged all tissue peaks into a peak list. The number of raw reads within each peak region was counted using bedtools coverage (bedtools v2.31.0). The raw count matrix was normalized by Counts Per Million (CPM), and the average expression of replicate samples was used for downstream analyses. For sfPSCs and spPSCs, at least two‐fold change was used to define special peaks. For SEFs, at least 16‐fold change was used to annotate peaks with ChIPseeker (1.38.0).^[^ ^78^, ^79^ ^]^ ATAC‐seq signal tracks in bigWig format were generated using bamCoverage. Peaks counts were summarized using computeMatrix (deepTools v3.3.0, RPKM) and visualized in IGV (v2.16.1). The motif enrichment analysis was performed using the findMotifsGenome.pl tool.

### Statistical Analysis

All experiments were performed with three biological and technical replicates. Graphical presentation and statistical analysis were performed with GraphPad Prism 8.0. The values reported in the graphs were represented as means ± standard deviations, and statistical significance was calculated with Student's two‐tailed t‐test: P‐value less than 0.05 was considered statistically significant and was displayed as ^*^ *P *< 0.05, ^**^ *P *< 0.01, and ^***^ *P *< 0.001.

## Conflict of Interest

The authors declare no conflict of interest.

## Author Contributions

J.Z., R.L., R.L., Q.Z., and Z.Z. contributed equally to this work. S.C. and J.H. conceptualized and supervised the project. J.Z. performed sfPSCs and spPSCs derivation, in vitro culture and characteristics analysis. S.C., R.L., M.Z., H.S, J.Y., F.Z., T.L., H.W., X.Z., J.Z. and Q.L. coordinated and collected sheep embryonic single cells. Q.Z. and S.G.build the scRNA‐seq library and R.L. performed bioinformatics analyses of the scRNA‐seq. H.X. performed ATAC‐seq analysis. M.W., Y.Y., T.C. and Y.Y. performed H&E analysis of the teratoma and chimeras. Y.Y., Z.Z., W.J., Y.W. and P.H. performed chimera assay, Z.F. and S.W. performed embryo cloning using sheep PSCs. X.C. drawn the schematic diagram. J.Z., R.L., J.H., and S.C. performed manuscript wrote reviewed and edited the final manuscript.

## Supporting information



Supporting Information

Supporting Tables

## Data Availability

The raw sequence data reported in this paper have been deposited in the Genome Sequence Archive (GSA) in the National Genomics Data Center, China National Center for Bioinformation/Beijing Institute of Genomics, Chinese Academy of Sciences (GSA: CRA018492) that are publicly accessible at https://ngdc.cncb.ac.cn/gsa.
