# Supplementary material for: Tracing and Capturing the Epiblast Pluripotency of Sheep Preimplantation Embryos
Source: Adv Sci (Weinh). 2025 Jun 30;12(36):e17764. doi: 10.1002/advs.202417764 (PMC12463017; doi:10.1002/advs.202417764)
Supplement: Supplementary file 1 — Supporting Information [file ADVS-12-e17764-s002.docx]

Supporting Information

# Tracing and Capturing the Epiblast Pluripotency of Sheep Preimplantation Embryos

*Jinying Zhang*,^1,8^ *Runbo Li*,^1,8^ *Ruijie Luo*,^2,8^ *Qiang Zhang*,^3,8^ *Zimo Zhao*,^1,8^ *Haishen Xu*,^1^ *Minglei Zhi*,^1^ *Wenjie Jiang*,^4^ *Meng Wang*,^1^ *Xinze Chen*,^1^ *Zhiqiang Feng*,^1^ *Yingjie Wang*,^1^ *Yuhan Yang*,^1^ *Pengcheng He*,^1^ *Hanyue Su*,^2^ *Tianzhi Chen*,^1^ *Shunxin Wang*,^1^ *Yixuan Yao*,^1^ *Jinghui Yang*,^2^ *Fan Zhao*,^2^ *Tingting Li*,^5^ *Haitao* *Wang*,^5^ *Xiaosheng Zhang*,^6^ *Jinlong Zhang*,^6^ *Qiuyue Liu*,^5^ *Shuai Gao*,^7^ *Yuchang Yao*,^4,^* *Jianyong Han*,^1,^* *and* *Suying Cao*^2,^*


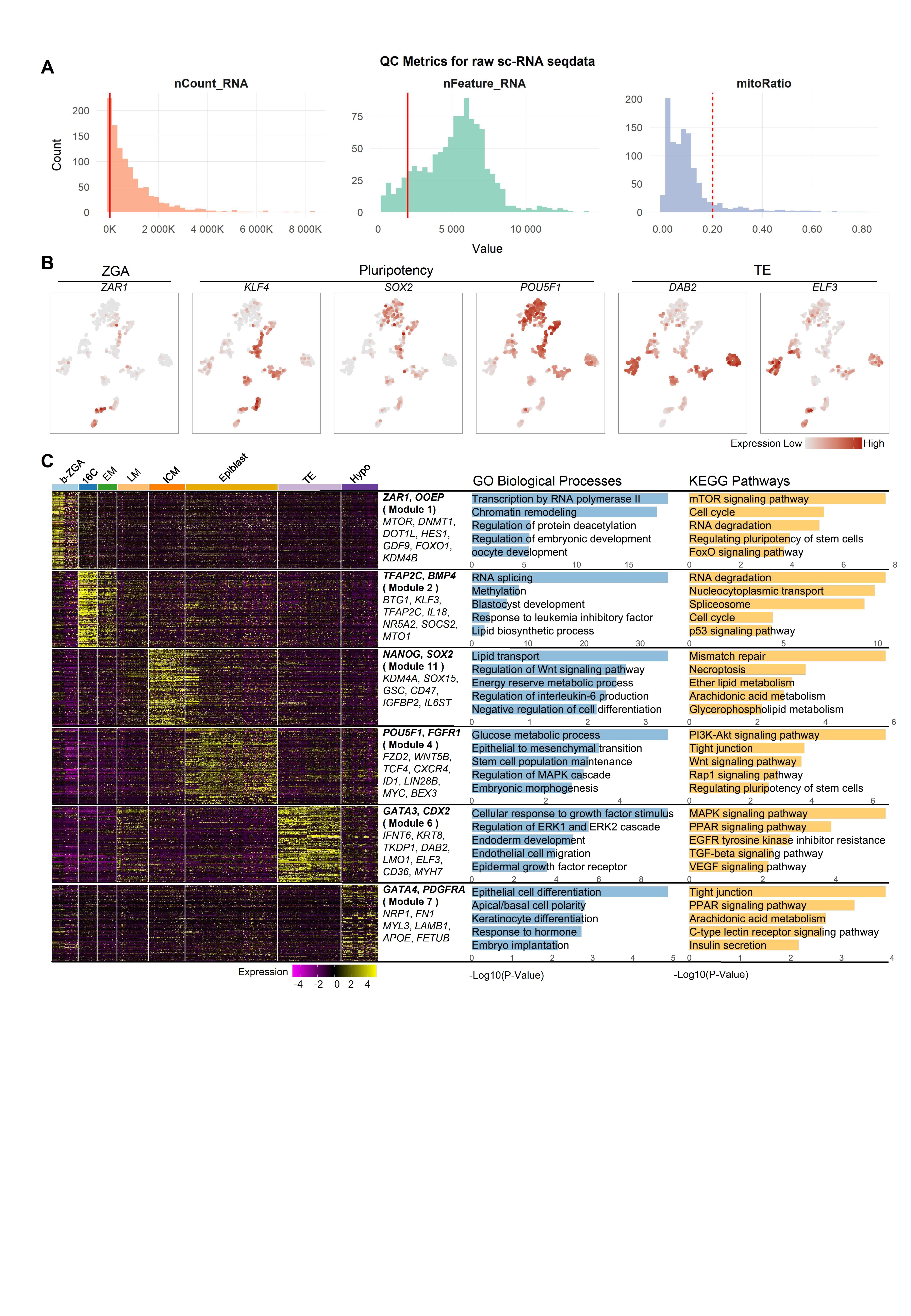


**Figure S1.** Single cell RNA-seq data quality control and cell type identification. A) Histograms display the distributions of quality control metrics for raw scRNA-seq dataset: (a) total UMI counts per cell (`nCount_RNA`), (b) number of expressed genes per cell (`nFeature_RNA`), and (c) proportion of mitochondrial gene expression (`mitoRatio`). Vertical red lines indicate filtering thresholds: solid lines denote lower limits (`nCount_RNA` ≥ 5,000, `nFeature_RNA` ≥ 2,000), and the dashed line indicates the upper limit for `mitoRatio` (≤ 0.2). These thresholds were used to select high-quality cells for subsequent analyses. B) t-SNE plots display the expression levels of different lineage specific marker genes of all cells, with a gradient from white to red indicating low to high expression. C) Gene co-expression module analysis of lineage specific genes. The heatmap displays gene modules with the highest correlation of different cell types. Representative genes and key GO terms and KEGG pathway enrichments are shown, a gradient from purple to yellow indicates low to high expression.


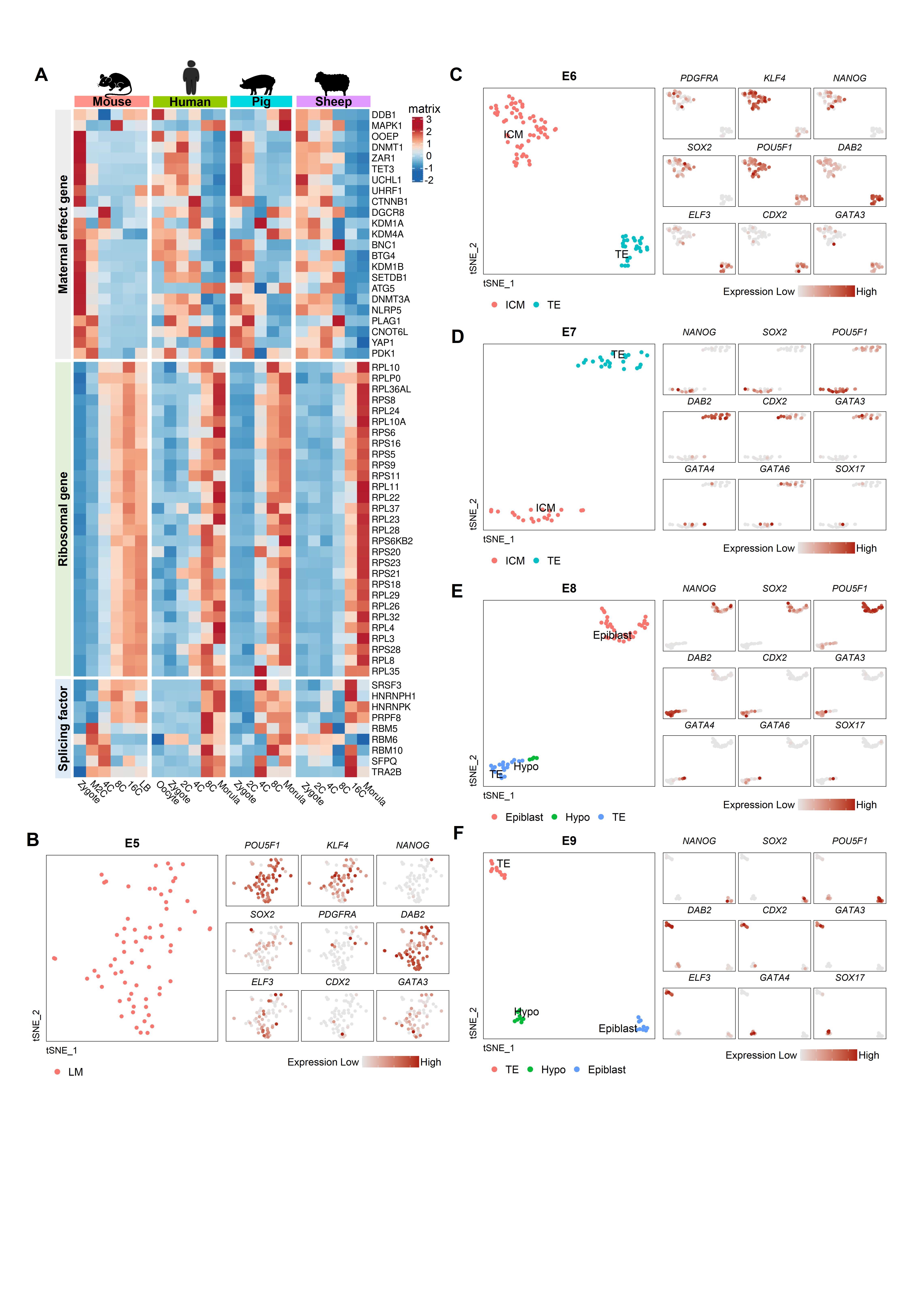


**Figure S2.** Identification the lineage specification of sheep embryos and comparison of ZGA development cross-species. A) Heatmap illustrates the expression patterns of maternal genes, ribosomal genes and splicing genes during ZGA development in mouse, pig, and sheep. B-F) t-SNE plots depict the clustering of cell types at each stage from E5 to E9, along with the expression levels of lineage specific marker genes. The color gradient from gray to red indicates an increase in gene expression, ranging from low to high.

**
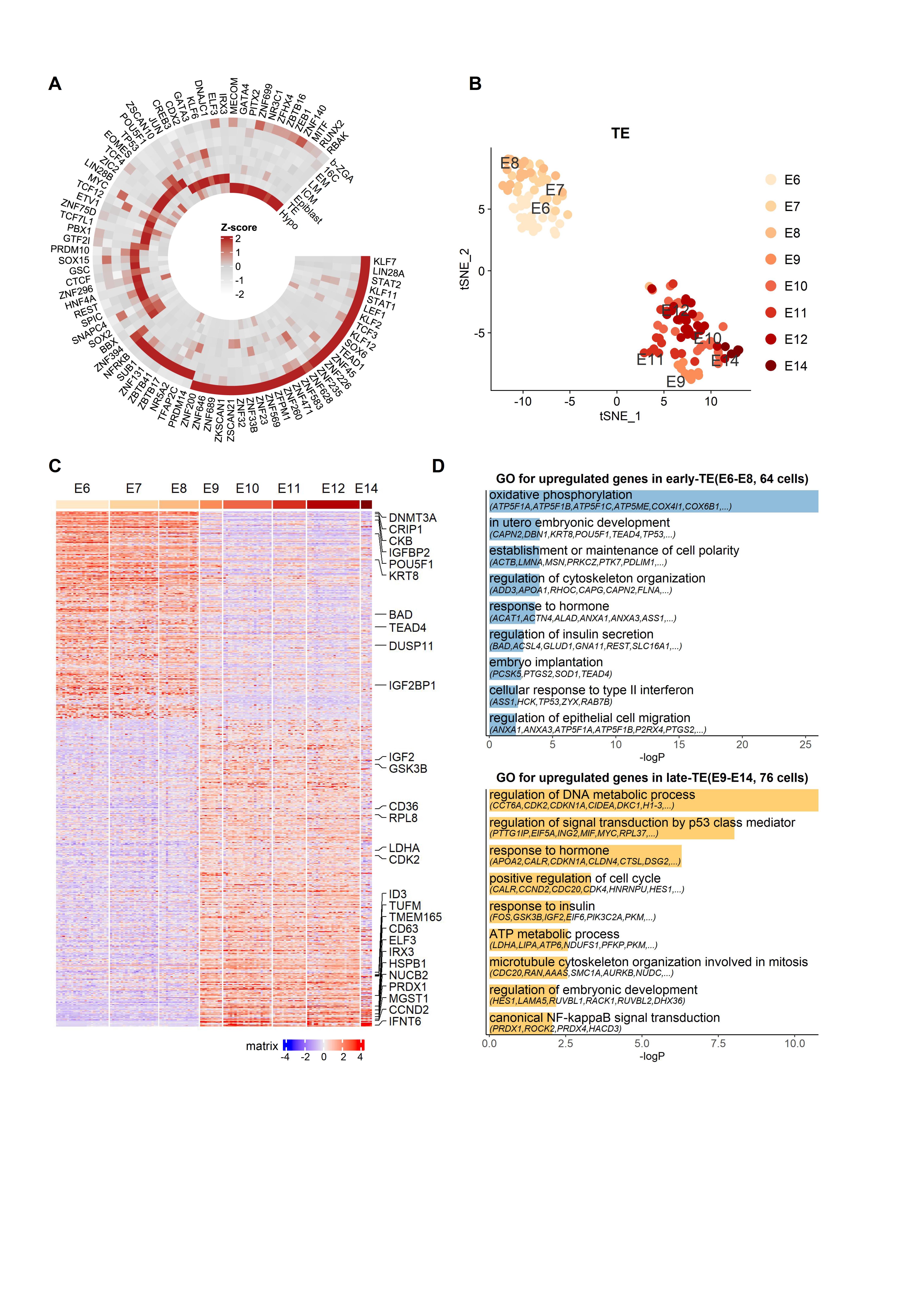
**

**Figure S3.** The profiling of sheep trophectoderm lineage development. A) Circular heatmap shows transcription factor expression across different stages and lineages in sheep embryos. B) t-SNE plots illustrate the distribution of TE cells at different embryonic stages. C) Heatmap showing the differential gene expression patterns associated with TE development from E6 to E14. D) GO enrichment analysis reveals the functional enrichment of differentially upregulated genes between early TEs (E6-E8) and late TEs (E9-E14).

**
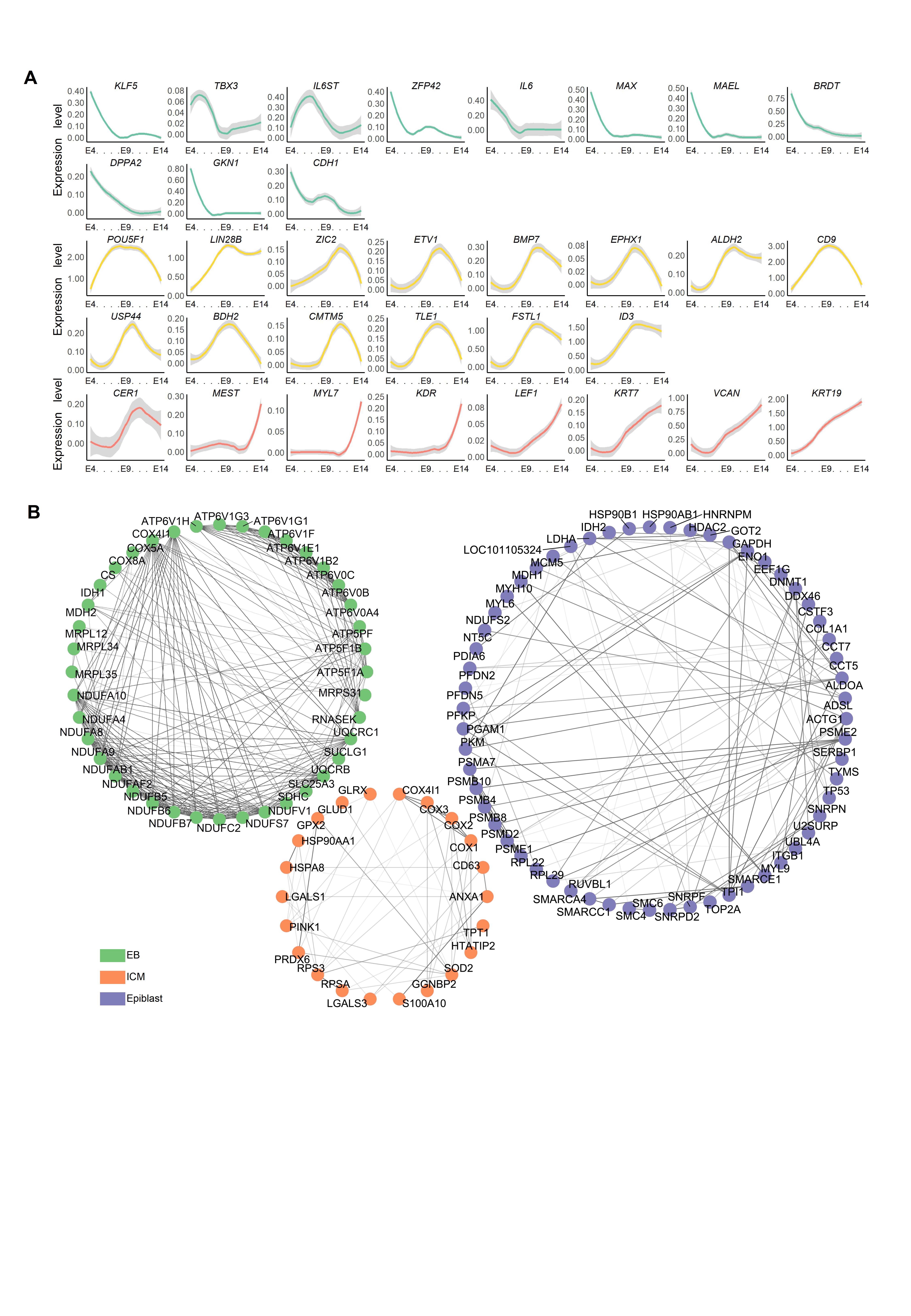
**

**Figure S4.** Gene expression dynamics and regulatory networks of sheep epiblast lineage. A) Expression trends of genes associated with naïve, formative, and primed states in epiblast lineage during E4 to E14. B) Construction of gene interaction network of differentially expressed genes in late morula (LM), inner cell mass (ICM), and epiblast cells using the STRING database. Nodes represent genes, with sizes scaled by expression levels, and edges indicate protein-protein interactions.


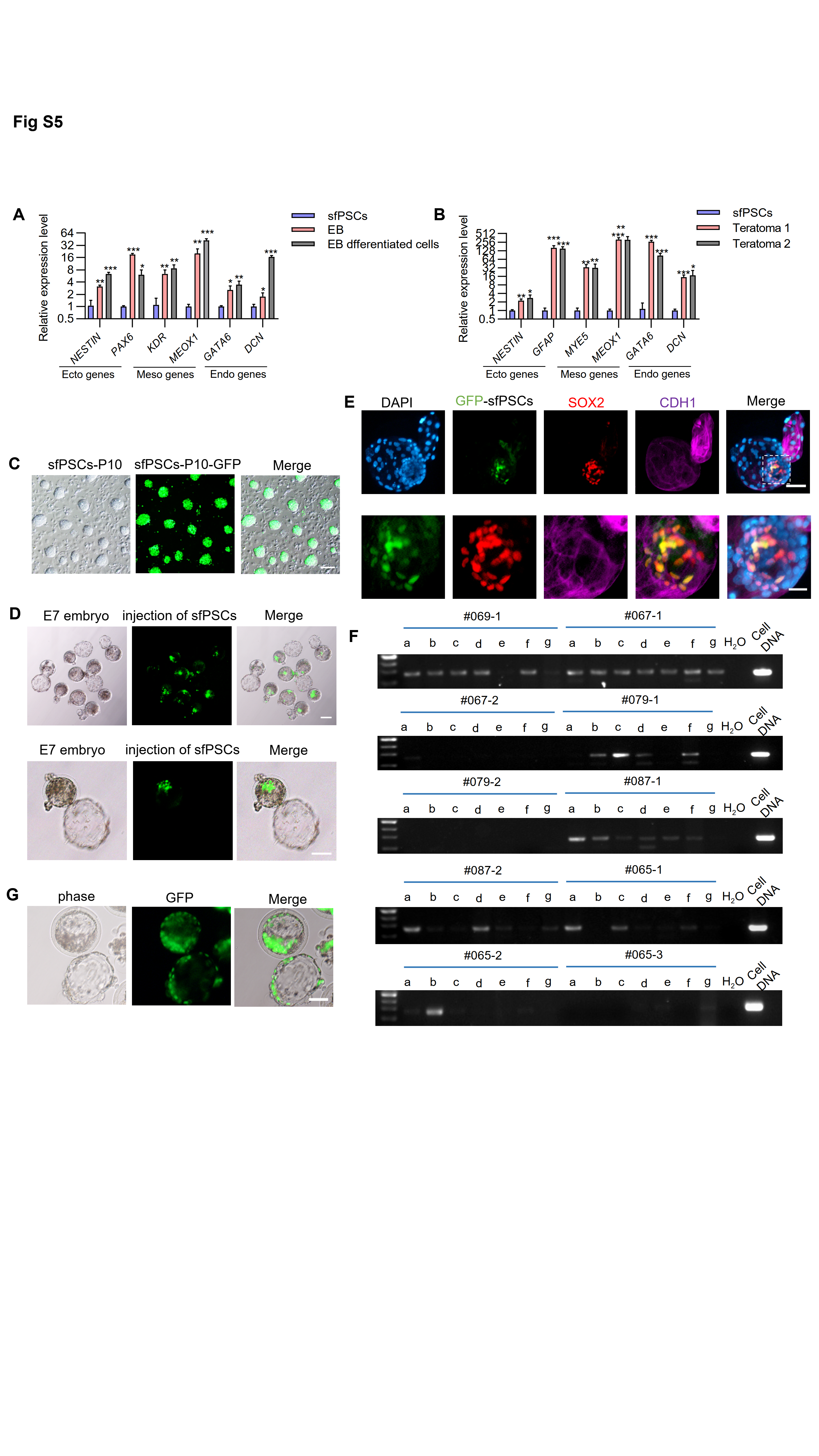


**Figure S5.** The development potential of sfPSCs. A) Expression analysis of three germ layer marker genes in EBs and EBs differentiated cells by RT-PCR. B) Expression analysis of three germ layer marker genes teratomas derived from sfPSCs by RT-PCR. C) Colony morphology of GFP-sfPSCs, scale bar, 100 μm. D) Morphological observation of chimeric embryos. Scale bar, 100 μm. GFP positive cells were detected in 91.38% of total 58 blastocysts (from 3 independent experiments were counted) produced by the injection assay. Analysis of the number of sfPSCs in chimeric embryos (n = 53) showed an average incorporation of (27.72 ± 8.63) GFP positive cells per embryo. E) Representative immunofluorescence images showing ICM incorporation of sfPSCs. DAPI (blue), sfPSCs (green), CDH1 (magenta) and SOX2 (red). Up, scale bar, 100 μm; bottom, scale bar, 25 μm. Similar results were obtained in three independent experiments. F) PCR genotyping for GFP DNA using the genomic DNA samples. Tissues of each embryo were isolated from head (a), trunk (b), heart (c), liver (d), intestines (e), kidney (f) and foetal membrane (g). G) Morphological of sfPSCs-SCNT embryos. Scale bar, 100 μm.


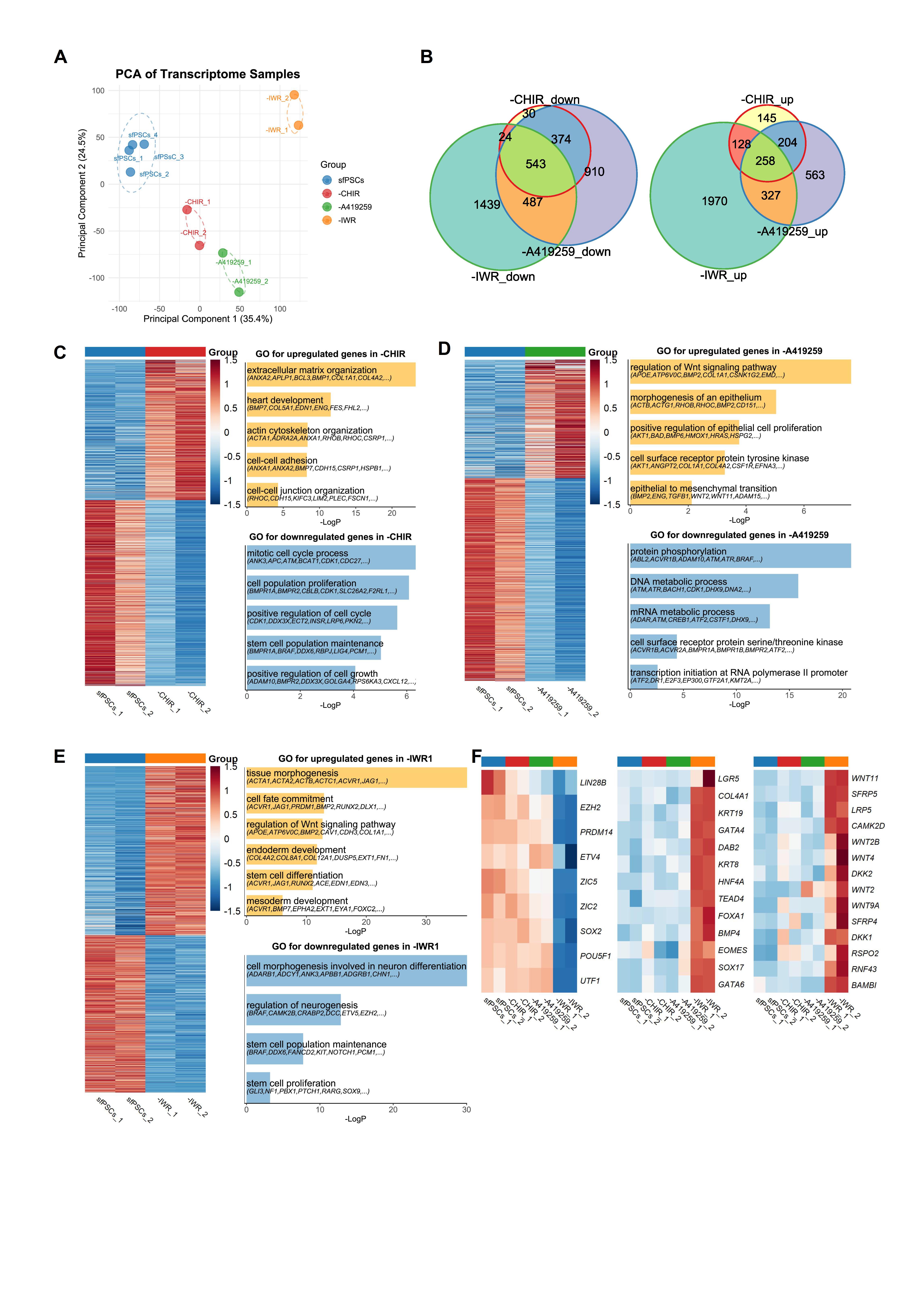


**Figure S6.** Transcriptomic analysis of the signaling components function after removal of CHIR99021, A419259 and IWR1 in the sfPSCs culture conditions. A) Principal component analysis (PCA) based on TPM values reveals global transcriptional differences among sfPSCs, -CHIR, -A419259, and -IWR1 groups. B) Differential expression analysis was performed for each comparison (CHIR vs sfPSCs, A419259 vs sfPSCs, IWR1 vs sfPSCs), identifying significantly up- and down-regulated genes (criteria: |log₂FC| > 1, FDR < 0.05). Venn diagrams depict the overlap and specificity of differentially expressed genes across comparisons. C-E) Enrichment analysis of up and down regulated pathways and representative genes in CHIR withdrawal (C), A419259 withdrawal (D), and IWR1 withdrawal (E) conditions compared to sfPSCs. F) Heatmap showing the expression patterns of selected pluripotency markers, differentiation related genes and WNT signaling genes across the four groups.


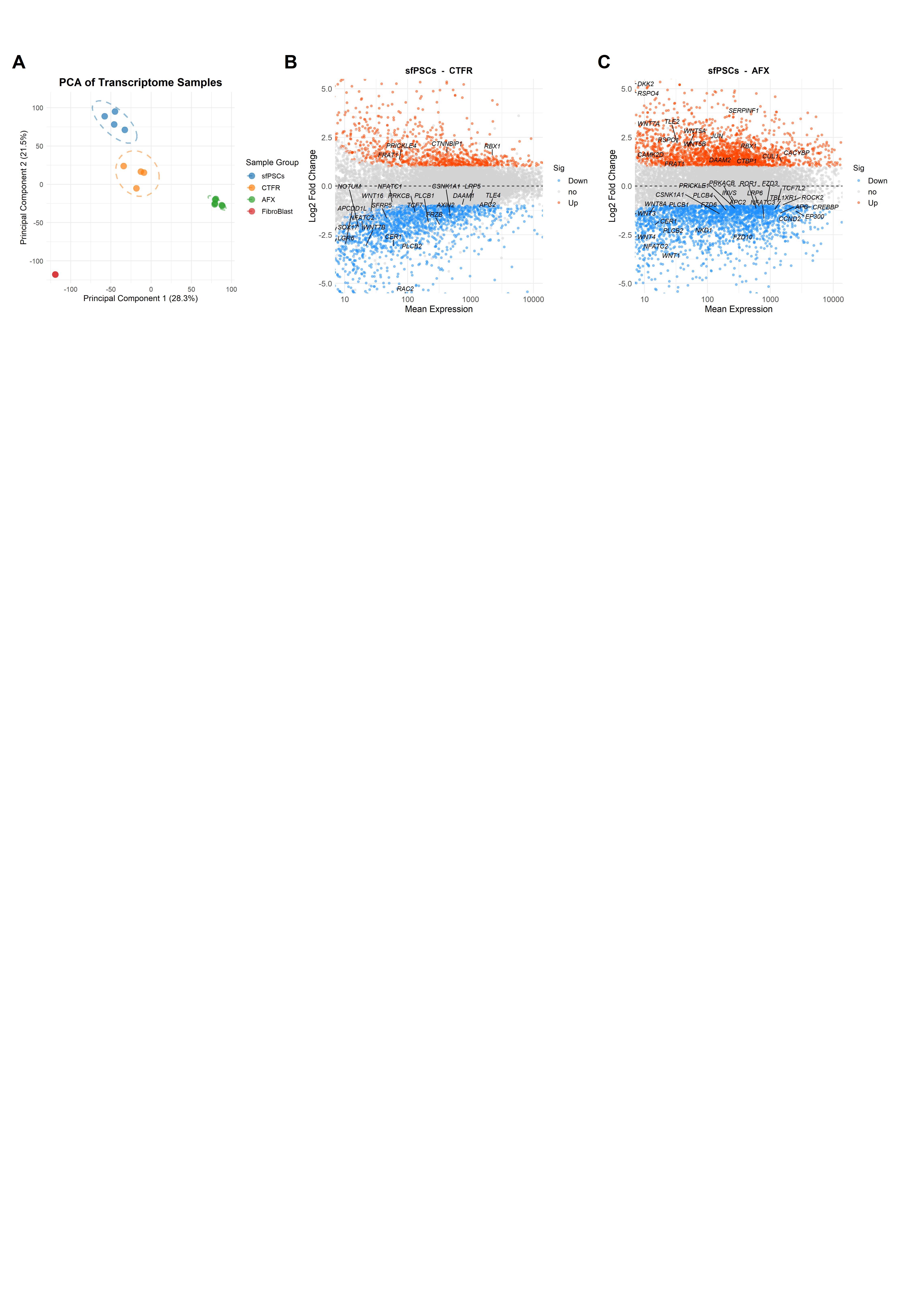


**Figure S7.** Comparative analysis of sfPSCs to previously reported sheep PSCs. A) Principal component analysis (PCA) of four groups: sfPSCs, CTFR, AFX, and Fibroblast. B-C) Volcano map plots showing the differentially expressed genes of sfPSCs, CTFR-PSCs (B) and AFX-PSCs (C).Members of the WNT signaling pathway genes are highlighted in the plots.


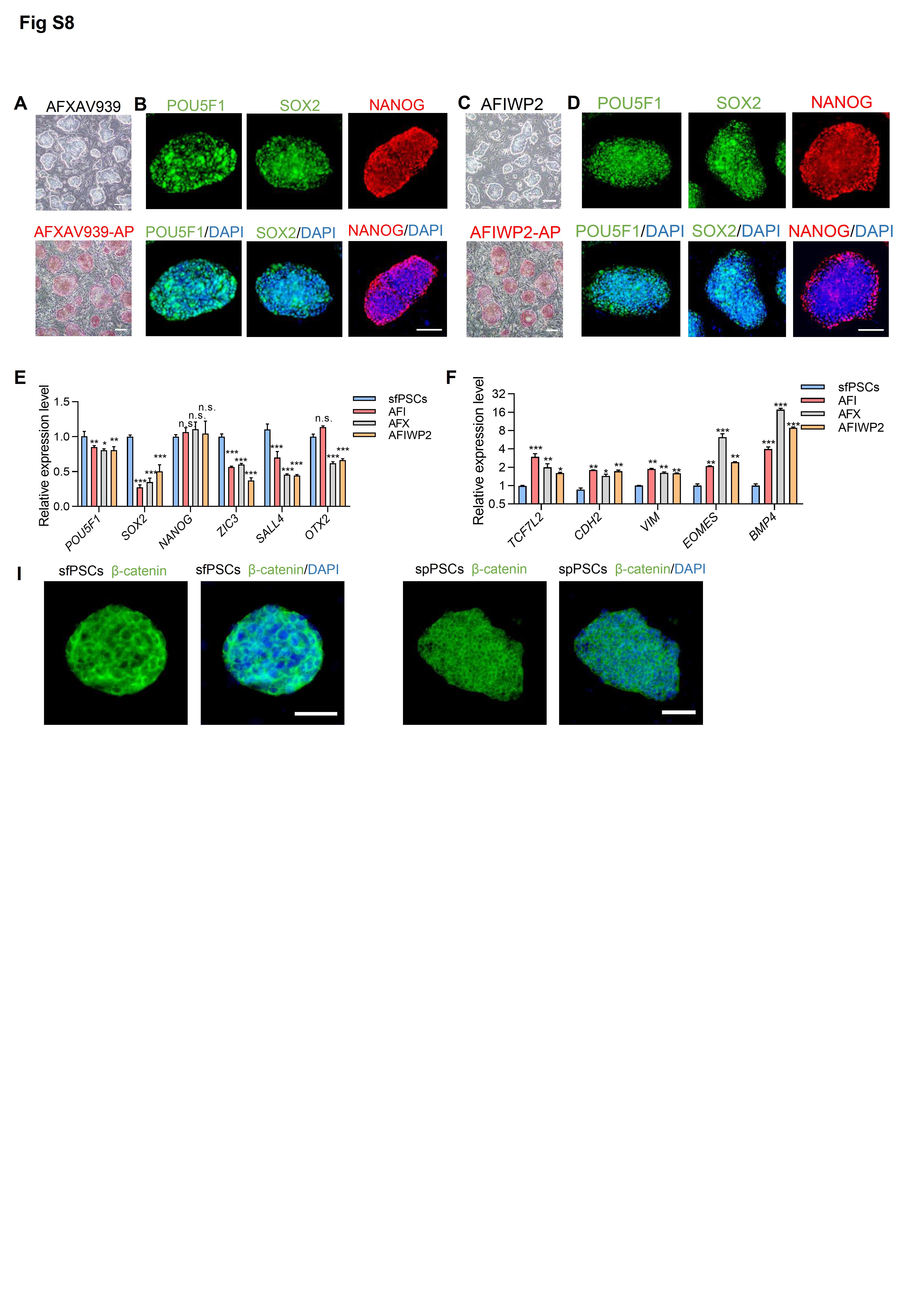


**Figure S8.** IWR1 could be replaced with other WNT inhibitors. A) Morphology and AP staining of spPSCs in AFXAV939 medium. Scale bar, 100 μm. B) Immunostaining of pluripotent markers POU5F1, SOX2 and NANOG of spPSCs in AFXAV939 medium, DAPI was used to stain nuclei. Scale bar, 50 μm. C) Morphology and AP staining of spPSCs in AFIWP2 medium. Scale bar, 100 μm. D) Immunostaining of POU5F1, SOX2 and NANOG of spPSCs in AFIWP2 medium, DAPI was used to stain nuclei. Scale bar, 50 μm. E) Representative pluripotent marker genes expression analysis in spPSCs cultured in different WNT inhibitor medium. F) Expression analysis of EMT and mesoderm marker genes in spPSCs under different culture conditions. For E, F, the error bar indicates ± SD (n = 3, independent experiments). I) Immunostaining of β-catenin protein of sfPSCs and spPSCs. Scale bar, 50 μm.


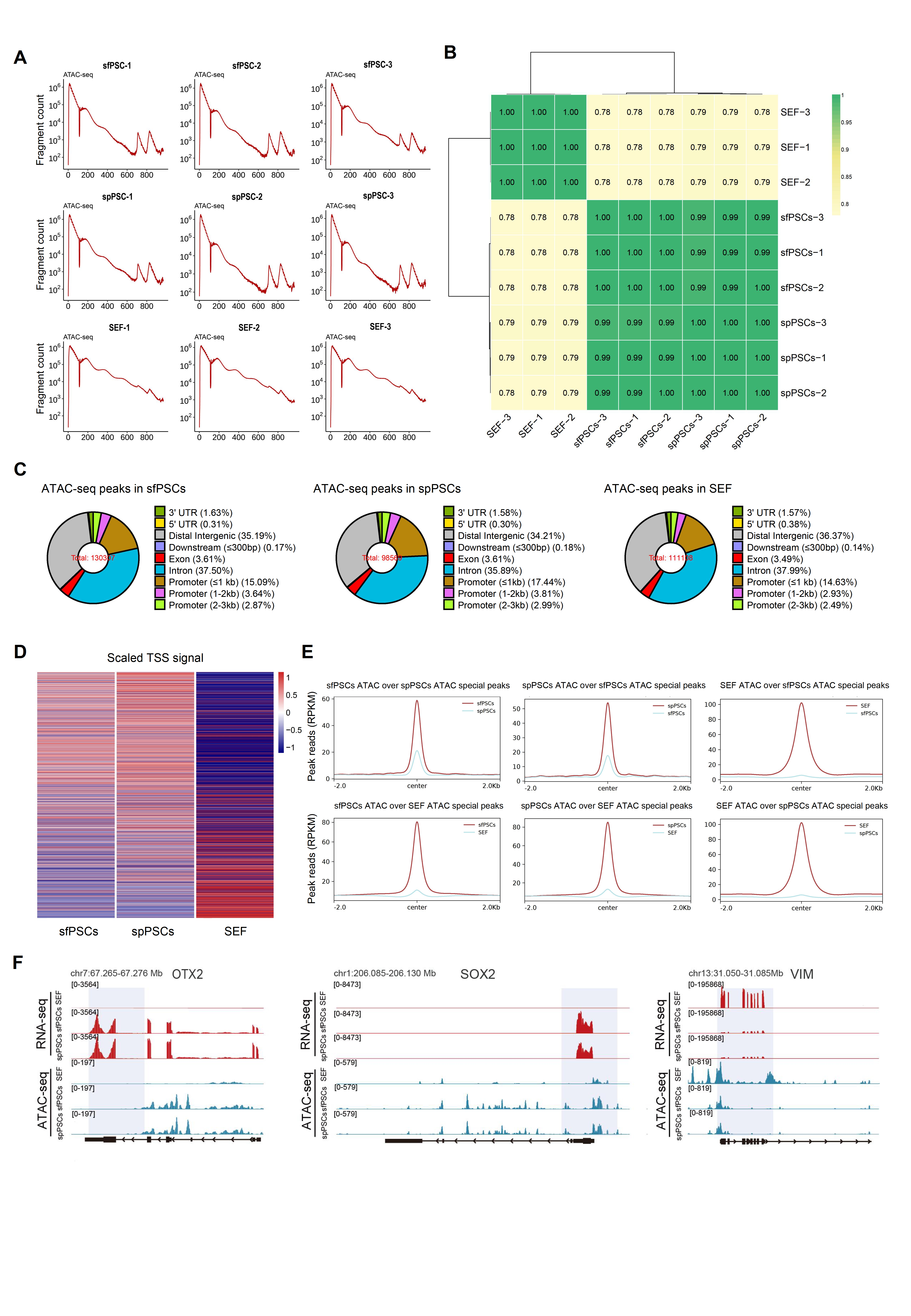


**Figure S9.** Analysis of chromatin accessibility in sfPSCs, spPSCs, and SEF. A) The size distribution of insert fragments within ATAC-seq libraries. B) Heatmap showing the correlation coefficients of nine ATAC-seq datasets reveals a high similarity among the biological replicates, as well as between spPSCs and sfPSCs (Pearson’s r = 1), while exhibiting more differently between PSCs and SEF (Pearson’s r < 0.8). C) Genome-wide annotation of open chromatin regions in sfPSCs, spPSCs and SEF. The peaks were divided into nine distinct categories, including promoters (i.e., promoter (≤ 1 kb), promoter (1-2 kb), promoter (2-3 kb), intron, exon, downstream, distal Intergenic, 3' UTR and 5' UTR. Notably, about 38 % peaks are localized in introns, the distal intergenic regions account for nearly 37%. The majority of peaks in promoters are primarily promoter (≤1 kb). D) The heatmap illustrates the distribution of reads across the region from the transcription start site (TSS) to the transcription end site (TES), extended by 2 kb respectively. E) ATAC-seq signals display cell type-sepcific peaks in sfPSCs, spPSCs and SEF (center, with extend to 2 kb upstream and downstream respectively). F) ATAC-seq and RNA-seq data tracks for the vicinity of transcription factors OTX2, SOX2 and VIM.
