# Supplementary material for: Tracing and Capturing the Epiblast Pluripotency of Sheep Preimplantation Embryos
Source: Adv Sci (Weinh). 2025 Jun 30;12(36):e17764. doi: 10.1002/advs.202417764 (PMC12463017; doi:10.1002/advs.202417764)
Supplement: Supplementary file 2 — Supporting Tables [file ADVS-12-e17764-s001.zip › Table S9.docx]

**Table S9.** RT-qPCR primer sequences used in this study.

| Gene name | Sequence (5’-3’) |  |
| --- | --- | --- |
| *BMP4* | F: ACCGAATGCTGATGGTCGTT | R: CAGAAGTGTGGCCTCGAAGT |
| *CDH2* | F: GGGATCAAAGCCGGGAACAT | R: GGCCATATGTGGGATTGCCT |
| *CER1* | F: CCGACAGCATGCCTCTTCTC | R: CTGGCACCTATCAGGTGTGG |
| *DCN* | F: AACAATATCTCTGCAATCGGCTC | R: AGTTTCCAAGCTGAACAGCAGC |
| *EOMES* | F: TTAGGGGAGCAGCTCTTGGT | R: AGAACTTCTTGGGCGCTTTG |
| *FOXA2* | F: CCCTTCTCCATCAACAACCT | R: GTAGGCCTTGAGGTCCATTT |
| *GAPDH* | F: ACGGGAAGCTCACTGGCATGG | R: GCCAGCCCCAGCATCGAAG |
| *GATA6* | F: CTCCTCTTCCTCCTGCTGCTCTC | R: ATGCGAGGCGTAGGGACTGAG |
| *GFAP* | F: CCTGCAGATCCGAGAAACCA | R: AAGAGACAGCTACCCCGACT |
| *ID2* | F: GACTTGCAGATCGCGCTAGA | R: TCAGAAGCCTGCAAGGACAG |
| *KDR* | F: GGGTCCTGAAATCACGTTGCA | R: AGTCTTCCTGTCCTGAGCAAAGC |
| *KLF4* | F:AGGGAGACGGAGGAGTTCAATGATC | R: AGGACGAGGAAGAGGCTGATGC |
| *MEOX1* | F: GGAGAATTCAGACAACCAGGAG | R: TGAGCAAACTCAGCTTCGAG |
| *MYE5* | F: GAATGCCATCCGCTACATTGAGAG | R: AATCCAGGTTGCTCTGAGTTGGT |
| *NANOG* | F: CAAGTATTTCAGTTCCCAGCAGCA | R: TCCCTCAAACTGACACAGAAGGTA |
| *NESTIN* | F: CACCTCAAGATGTCCCTCAGC | R: TCTTCAGAAAGGTTGGCACAG |
| *OTX2* | F: AGCAAATCTCCCTGAGAGCG | R: GGGTTTGGAGTGGTGGAACTTA |
| *PAX6* | F: CCCTGGAGAAAGAGTTTGAGAG | R: TCCATTTGGCCCTTCGATTAG |
| *POU5F1* | F: TACACTGTACTCTTCGGTCCCATT | R: AGCATCATTGAACTTCACCTTCCC |
| *SALL4* | F: CACAAGTGTCGGAGCAGTGT | R: GCTGCTAACAGAGGCGTCAT |
| *SOX2* | F: TACGGTAGGAGCTTTGCAGAAAGT | R: TGCACGTTTGCAACTGTCCTAAAT |
| *STAT3* | F: CTGAGAGAGCAGAGATGCGG | R: ACGTTCTTGGGGTTGTTGGT |
| *TCF7L2* | F: CTTGCTGCACTCAGGGACAT | R: GCCGTGGGATTCCTGTTTTG |
| *VIM* | F: CGTGATGTCCGTCAGCAGTA | R: GTCGTTGTTGCGGTTAGCAG |
| *ZIC3* | F: AGGCTGTGACAGACGCTTTG | R: CGGGTGGAGTGGAAGATTCAT |
| *GFP-*Test | CAACACCCGCATCGAGAAGT | ACCACGAAGCTGTAGTAGCC |
