# Supplementary material for: Tracing and Capturing the Epiblast Pluripotency of Sheep Preimplantation Embryos
Source: Adv Sci (Weinh). 2025 Jun 30;12(36):e17764. doi: 10.1002/advs.202417764 (PMC12463017; doi:10.1002/advs.202417764)
Supplement: Supplementary file 2 — Supporting Tables [file ADVS-12-e17764-s001.zip › Table S8.docx]

| Antigen | Company | Cat No. | Dilution |
| --- | --- | --- | --- |
| Anti-OCT-3/4 | Santa Cruz Biotechnology | Cat# sc-5279 | 1:500 |
| Anti-SOX2 | Santa Cruz Biotechnology | Cat# sc-365823 | 1:500 |
| Anti-NANOG | PeproTech | Cat# 500-P236 | 1:500 |
| Anti-SSEA1 | Abcam | Cat# ab16285 | 1:300 |
| Anti-SSEA4 | Abcam | Cat# ab16287 | 1:300 |
| TRA-1-60 | Cell Signaling Technology | Cat# 4746 | 1:300 |
| Anti-NESTIN | Abcam | Cat# ab81755 | 1:300 |
| Anti-α-SMA | Abcam | Cat# ab5694 | 1:300 |
| Anti-GATA6 | Abcam | Cat# ab22600 | 1:300 |
| Anti-beta III Tubulin | Abcam | Cat# ab18207 | 1:300 |
| Anti-H3K27me3 | Active motif | Cat# 39155 | 1:400 |
| Anti-β-Catenin | Santa Cruz Biotechnology | Cat# sc-7963 | 1:200 |
| Goat anti-Mouse IgG (H+L) Cross-Adsorbed Secondary Antibody, Alexa Fluor 488 | Thermo Fisher Scientific | Cat# A-11001 | 1:1000 |
| Goat anti-Mouse IgG (H+L) Cross-Adsorbed Secondary Antibody, Alexa Fluor 594 | Thermo Fisher Scientific | Cat# A-11005 | 1:1000 |
| Donkey anti-Rabbit IgG (H+L) Cross-Adsorbed Secondary Antibody, Alexa Fluor 594 | Thermo Fisher Scientific | Cat# A-21207 | 1:1000 |
| [Goat anti-Chicken IgY (H+L) Secondary Antibody, Alexa Fluor™ 488](https://www.thermofisher.cn/antibody/product/Goat-anti-Chicken-IgY-H-L-Secondary-Antibody-Polyclonal/A-11039) | Thermo Fisher Scientific | Cat# A-11039 | 1:1000 |
| Anti -Phospho-Stat3 (Tyr705) | Cell Signaling Technology | Cat# 52075 | 1:500 |
| Anti -GAPDH (D16H11) | Cell Signaling Technology | Cat# 5174 | 1:1000 |
| Anti-rabbit IgG, HRP-linked Antibody | Cell Signaling Technology | Cat# 7074 | 1:5000 |

**Table S8.** Antibodies used in this study.
